# Polyurethane Foam Emission Samplers to Identify Sources of Airborne Polychlorinated Biphenyls from Glass-Block Windows and Other Room Surfaces in a Vermont School

**DOI:** 10.1021/acs.est.3c05195

**Published:** 2023-09-15

**Authors:** Jason B. X. Hua, Rachel F. Marek, Keri C. Hornbuckle

**Affiliations:** Department of Civil and Environmental Engineering, IIHR-Hydroscience and Engineering, The University of Iowa, Iowa City, Iowa 52242, United States

**Keywords:** PUF-PES, semivolatile organic compounds, persistent organic pollutants, legacy compounds

## Abstract

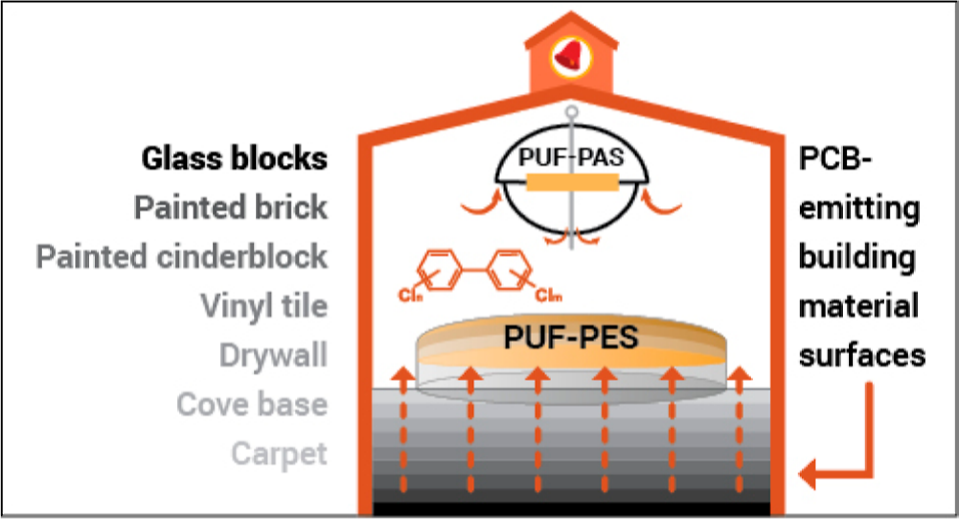

We hypothesized that emissions of polychlorinated biphenyls (PCBs) from Aroclor mixtures present in building materials explain their concentrations in school air. Here, we report a study of airborne concentrations and gas-phase emissions in three elementary school rooms constructed in 1958. We collected airborne PCBs using polyurethane foam passive air samplers (PUF-PAS, *n* = 6) and PCB emissions from building materials using polyurethane foam passive emission samplers (PUF-PES, *n* = 17) placed over flat surfaces in school rooms, including vinyl tile floors, carpets, painted bricks, painted drywall, and glass-block windows. We analyzed all 209 congeners represented in 173 chromatographic separations and found that the congener distribution in PUF-PES strongly resembled the predicted diffusive release of gas-phase PCBs from a solid material containing Aroclor 1254. Concentrations of airborne total PCBs ranged from 38 to 180 ng m^–3^, a range confirmed by an independent laboratory in the same school. These levels exceed action levels for all aged children set by the State of Vermont and exceed guidance levels set by the U.S. EPA for children under age 3. Emissions of PCBs from the glass-block windows (30,000 ng m^–2^ d^–1^) greatly exceeded those of all other surfaces, which ranged from 35 to 2700 ng m^–2^ d^–1^. This study illustrates the benefit of the direct measurement of PCB emissions to identify the most important building remediation needed to reduce airborne PCB concentrations in schools.

## Abstract 2

We found Aroclor 1254 emissions from glass-block windows to be a dominant source of airborne polychlorinated biphenyls in an elementary school.

## Introduction

Polychlorinated biphenyls (PCBs) were widely used in the United States for a variety of industrial and commercial applications because of their stability and longevity. Their persistent nature and widespread use present health risks and environmental contamination. Consequently, production of PCBs was banned in the 1970s–1980s in most industrial countries. Despite being banned, PCBs are still present in the environment and continue to pose a threat to human health.

Schools are a place of particular concern for PCB contamination and exposure. The period of PCB manufacturing (1930–1977) coincided with the period of active school construction in the United States (1950–1980). Approximately 55,000 schools in the United States, both public and private, were constructed in this era.^1^ In schools, PCBs were used in light ballast capacitors and building materials such as caulking and adhesives.^2,3^ Emissions of PCBs from these materials in schools may pose a significant risk of inhalation exposure to occupants, especially children.^4–8^

The United States Environmental Protection Agency (U.S. EPA) derived health protective levels for evaluation of airborne PCBs in schools based on age groups that range from 100 to 500 ng m^–3^. The EPA does not require schools to test for PCBs, nor does the EPA have enforceable threshold air concentrations that require schools to remediate. In 2020, the State of Vermont set a screening level (15 ng m^–3^) for PCBs in school air based on cancer and noncancer health effects, and in 2021, the state set regulatory action levels (Table 1) and passed legislation requiring schools built or renovated before 1980 to test for airborne PCBs using low-volume polyurethane foam (PUF) sampling (U.S. EPA Method TO-10).^9–13^

**Table 1:** Vermont Regulations of PCBs in School Air

	school action levels (ng m^–3^)	immediate action levels (ng m^–3^)
pre-K	30	90
K–6th grade	60	180
7th grade–adult	100	300

Few studies in the peer-reviewed literature have reported concentrations of airborne PCBs in schools, although districts in California, Massachusetts, New York City, and Vermont reported finding high airborne PCB concentrations that resulted in action, including demolition and replacement of the schools.^14^ In Germany, a study reported concentrations of 1000 ng m^–3^ and higher in 8 rooms of a school.^15^ In Denmark, Brauner et al. reported concentrations in 11 schools to be higher than 1000 ng m^–3^.^16^ In the United States, Ampleman et al. (2015) were the first to report that concentrations in schools exceeded PCB concentrations in the same students’ homes.^17^ Marek et al. (2017) and others report that for the same cohort of children, exposure to school air was comparable to exposure to PCBs in their diet.^18,19^ Bannavti et al. (2021) found that airborne PCBs in schools varied from room to room in a school, indicating the potential for localized sources in building materials.^20^

It is critical to identify those building materials that contribute to the highest PCB air concentrations, so the materials can be remediated to reduce inhalation exposure. Although no federal requirement exists to test school building materials for PCBs except during remodeling, mitigation, or demolition, PCBs found above 50 ppm in these materials are required to be removed and disposed of as hazardous materials.^21^ Yet the potential for PCB emissions from solid materials is not well-defined. It is possible that PCB emissions from materials containing less than 50 ppm cause exceedances of Vermont regulations or EPA guidelines for PCBs in school air. It is also possible that materials containing 50 ppm or more do not emit PCBs. To begin to address this knowledge gap, researchers from the U.S. EPA assessed the emissions from PCB-containing light ballasts and caulk, using chamber studies.^22,23^ While providing valuable information regarding the emission potential of two common building materials, these studies relied on destructive sampling and specialized chambers. In addition, removing materials from their location in the school may alter the material surface area and composition of PCBs that are likely to volatilize. Therefore, the in situ measurement of PCB emissions is a more effective method for directly assessing the contribution of various materials to PCBs in school air.

The distribution of PCB congeners provides important information about the original source, potential weathering, and differential release of PCBs from solid surfaces to air. For example, we have previously shown that statistical analysis of congener signals can distinguish airborne PCBs arising from Aroclor emissions from non-Aroclor emissions in schools.^20,24^

Here, we report the first measurements of PCB emissions from several different types of surfaces in a school. We measured emissions using polyurethane foam passive emission samplers (PUF-PES), a nondestructive, in situ method. The PUF-PES were placed over flat surfaces in schoolrooms, including tile flooring, carpet, painted brick, painted drywall, and glass-block windows. We also placed polyurethane foam passive air samplers (PUF-PAS) and analyzed all of the samples for 209 PCBs. We hypothesized that there are local sources in schoolrooms that explain, through measurements and modeling, the concentrations and variability of PCBs throughout a school. We also hypothesized that the congener signal provides evidence of the use of specific historical Aroclor mixtures purposefully placed in the school. In this study, we examined the performance and effectiveness of the simultaneous deployment of these samplers to identify the source of the most important emissions in a schoolroom.

## Methods

### Sampler Deployment

We collected samples at an elementary school in southeast Vermont in the summer of 2022 when no students were present. This school was among the first to be selected for airborne PCB testing by the State of Vermont based on prioritization factors including year of construction or renovation, completion of PCB mitigation, planned HVAC updates, planned construction, age of students, and free and reduced lunch percentages. Prior air sampling results indicated that some rooms exceeded Vermont’s action levels. Bulk sampling results of materials across the school also exceeded the 50 ppm level for action defined by the Toxics Substances Control Act.^21^ Our team was invited to assist the Vermont Department of Environmental Conservation (DEC) and the school to identify the cause of the high airborne PCB concentrations in room 203.

**Figure 1:**
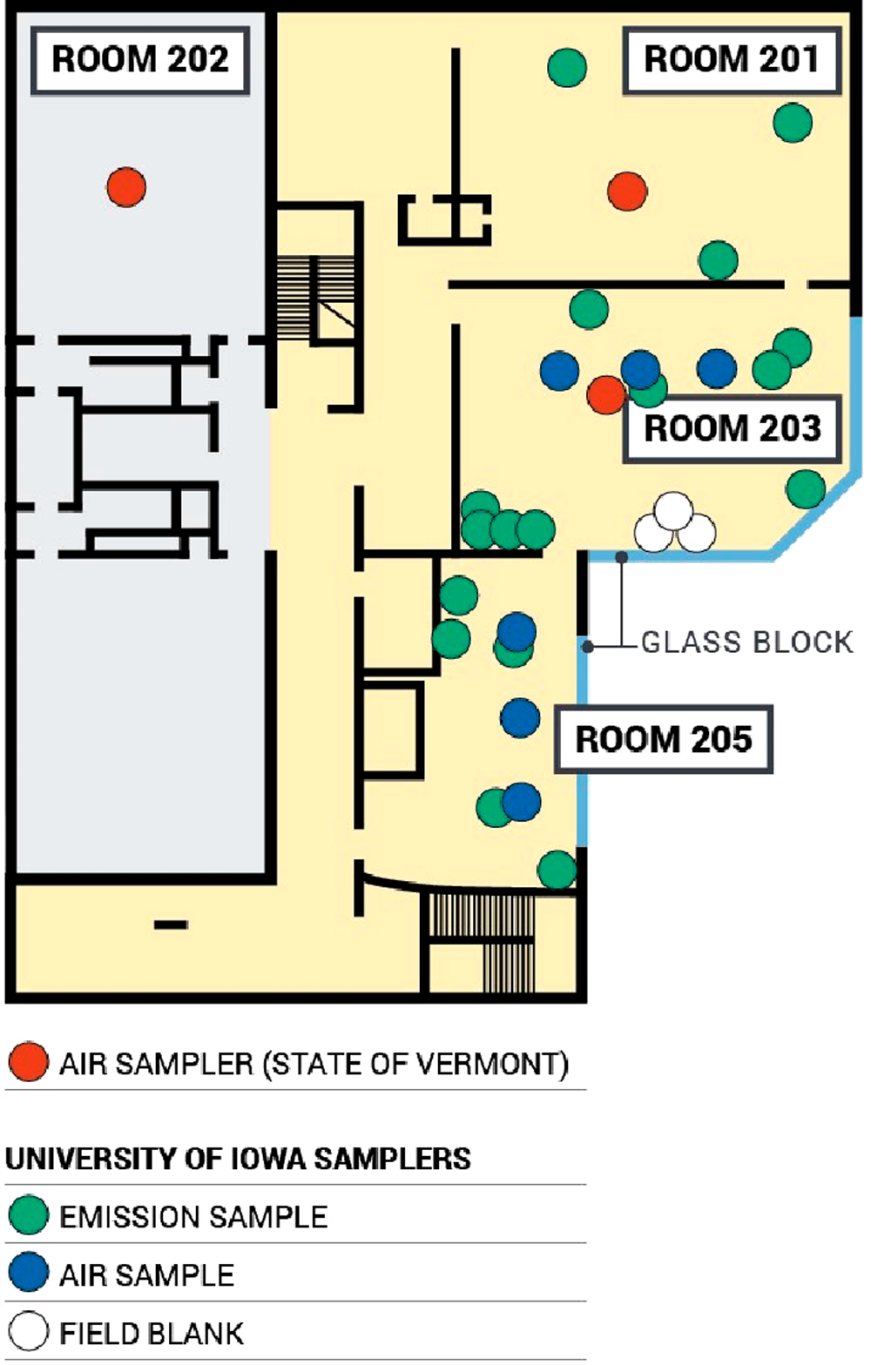
PUF-PAS, PUF-PES, and field blanks were deployed in three classrooms (201, 203, and 205) for 34 days. In a separate study reported by the State of Vermont, low-volume PUF samplers were placed in classrooms (including 201, 202, 203, and 112, not shown) and a hallway for 24 h during the same sampling period. Yellow areas were constructed in 1958 and grey areas were constructed in 1919.

We sampled airborne PCBs in two rooms and PCB emissions in three adjacent classrooms (Figure 1). According to school officials, these rooms have been used as schoolrooms since their construction in 1958. All three schoolrooms were furnished with desks, chairs, bookshelves, and cabinets, and each served ∼25 students every weekday during the regular school year (September through June). Two of the rooms included approximately 9 m^2^ area of glass blocks above the windows on an outside wall. Each room was connected via doorways within the rooms as well as a hallway. The floors in each room were carpeted. Two classrooms also had exposed vinyl tiles along one side of the room. Airflow between rooms was limited to passive movement through shared doors. Each room was equipped with unit ventilators that remained off for the duration of this study. The doors in these three rooms were closed, and the rooms were unoccupied throughout the deployment period.

We collected airborne PCBs using Harner-style double-dome PUF-PAS.^25^ We placed three PUF-PAS in room 203 and three in room 205. In brief, the PUF-PAS consists of two inverted stainless-steel bowls with an aluminum connecting rod that holds a disk of PUF and retains a gap for airflow. The PUF disks were cleaned prior to deployment using pressurized acetone and hexane solvent (Dionex ASE 350), dried, and tightly wrapped in foil until deployment. The samplers were hung from overturned desks at a height of 1.5 m. The samplers collected airborne PCBs for 34 days, and then the PUF material was removed, wrapped in aluminum foil, and placed in labeled Ziploc bags. The samples were extracted and quantified as described below. The concentration of each individual or coeluting congener in air was calculated as the mass of each PCB congener divided by the effective sampling volume.

The effective sampling volume, *V*_eff_, is a congener-specific function of the hourly sampling rate for the PUF-PAS, indoor wind speeds, room temperature, the air/PUF partition ratio (*K*_PUF_, unitless), the PUF volume (*V*_PUF_, m^3^), the deployment time (*d*, days), and the sampling rate (*R*_s_, m^3^ d^–1^).^26^ The indoor sampling rate is calculated as a function of the indoor windspeed when the ventilation is on or off (WS_on_ and WS_off_, m s^–1^), the fraction of the day ventilation is on or off (*f*_on_ and *f*_off_, unitless), the molecular weight of each congener, the air temperature (*T*, °C), and an empirical constant of the air samplers (c, 1.326). For this PUF-PAS deployment period, PCB uptake was in the linear phase for most congeners, and the integrated average of *R*_s_ ranged from 1.1 (PCB1) to 0.78 m^3^ d^–1^ (PCB209) with an average of 0.90 m^3^ d^–1^. Only congeners with one chlorine approached equilibrium with the room air. Details of this calculation are provided in the Supporting Information.

To calculate the sampling rate and effective volume, we selected parameters relevant to our sampling site and deployment time (Table 2). Room temperatures were measured in rooms 203 and 205 to estimate the average temperature over the deployment period (TSI Q-TRAK 7575) and averaged 30 °C. Higher temperatures increase the sampling rate and therefore increase the effective volume. We assumed *f*_off_ = 1. The mean wind speed was estimated from previously reported measurements.^26^ PUF-PAS effective sampling volumes and sampling rates for the 34 day deployment period were congener-specific. The effective sampling volume ranged from 23 m^3^ for 2-chlorobiphenyl (PCB1) to ∼40 m^3^ for congeners with two or more chlorine atoms and are reported in the Supporting Information. Congener-specific uncertainty analysis was previously reported for this method.^20^

**Table 2:** Parameters Used for Calculating the Effective Volume and Sampling Rate

parameter	value
deployment time	34 (days)
windspeed (off)	0.07 (m s^–^^1^)
fraction (off)	1 (unitless)
average temperature	30 (°C)
gas constant	8.314 (J K^–^^1^ mol^–^^1^)
sampler constant	1.326 (unitless)
PUF surface area	1.53 × 10^–^^2^ (m^2^)
PUF volume	2.3 × 10^–^^4^ (m^3^)

**Figure 2:**
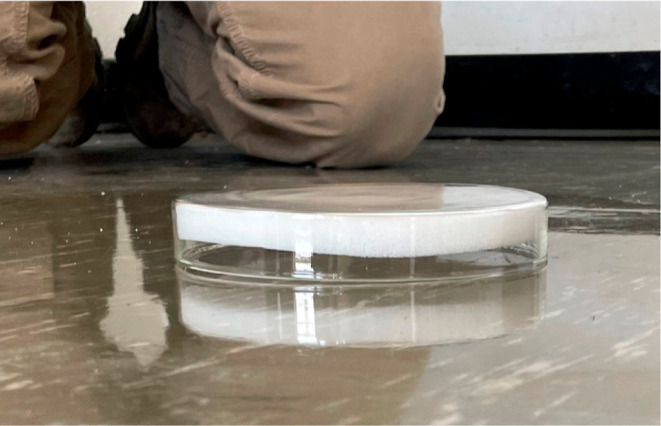
Polyurethane foam passive emission sampler (PUF-PES) is affixed to the surface, leaving an air gap, ensuring gas-phase collection of emitted PCBs.

We placed 17 PUF-PES on flat surfaces throughout the three rooms. We selected potential emission sites that represented the major exposed surfaces in the room. The PUF-PES design has been previously described and consists of a clean PUF disk tightly held in the bottom of a glass Petri dish (14 cm diameter, 2 cm depth).^27,28^ The PUF-PES was placed over a flat surface and then covered with foil and duct taped in place. Because the PUF sits tightly in the Petri dish that is about 5 mm deeper than the PUF thickness, there is an air gap between the PUF disk and the surface, assuring that PCBs are captured after volatilization, rather than direct contact with the surface (Figure 2). PUF field blanks were transported to the site but not opened. They were labeled and taped near a corresponding air or emission sample and remained on-site for the deployment period. At the end of the deployment period, samples were collected, wrapped in foil, and shipped back to the University of Iowa for analysis. The samples were extracted and quantified as described below. The emission of each individual or group of coeluting congeners was calculated as the mass of each PCB congener divided by the surface area covered by the PUF-PES and the deployment time, *t*. The total PCB emission rate is the sum of the 173 individual and coeluting congeners.

### PCB Extraction and Analyses

PUF was extracted using a pressurized and heated solvent as reported elsewhere and detailed in the Supporting Information.^20^ In brief, samples were spiked with a surrogate standard solution (13C labeled PCBs 3, 15, 31, 52, 118, 153, 180, 194, 206, and 209), extracted with a heated, pressurized 1:1 solvent mixture of hexane and acetone (Thermo Fisher Scientific Dionex ASE 350), and concentrated from 60 to 1 mL under nitrogen stream (Biotage TurboVap II). The extracts were then passed through acidified silica gel columns and concentrated to 0.5 mL. Internal quantification standards (d-PCB 30, PCB 204) were injected, and the extract was analyzed by gas chromatography with triple quadrupole mass spectrometry (Agilent 7000B Triple Quad with Agilent 7890A GC) in multiple reaction monitoring mode. This method results in the mass determination of 173 individual or coeluting congener peaks.

We assessed the quality of our data by considering measures of accuracy, precision, reproducibility, representativeness, and comparability. A limit of quantification (LOQ) was calculated as the upper limit of the 99th confidence interval of the log 10-transformed blank masses: average congener mass in blank PUF samples plus 2.325 times the standard deviation divided by the square root of *n* (Supporting Information). Method blanks (*n* = 4) and field blanks (*n* = 3), which approximated a log-normal distribution, are included in the calculations of the LOQ. Congener masses are reported as measured and not replaced with different values when below LOQ. LOQ values ranged from 0.05 to 3.03 ng per congener. The accuracy of our methods was assessed using standard reference material analysis of certified PCB concentrations in house dust sprinkled on PUF (NIST, SRM 2585, Gaithersburg, MD, USA). To assess reproducibility and precision, carpet emission samples were deployed in triplicate in each room. The precision of our extraction method was assessed with surrogate standards and method blanks. The average sum of the method blank and field blank was 4.2 and 6.4 ng, respectively. The surrogate recoveries ranged from 36 to 103%. We corrected sample masses for surrogate recoveries below 100%. The full data set of congener-specific measurements and quality control assessment has been released to an open-access data repository.^29^ Additional details of our method can be found in the Supporting Information.

## Results and Discussion

Concentrations and emissions of airborne PCBs were determined for each of the 173 congener or coeluting congener groups and the sum of the PCBs (ΣPCB) (Table 3). We found concentrations of ΣPCB to range from 38 to 180 ng m^–3^. The average concentrations in room 203 (*n* = 3) and room 205 (*n* = 3) were 140 ± 43 and 43 ± 7 ng m^–3^, respectively. These concentrations were much higher than those reported for urban outdoor environments around the world.^25^ Our findings are comparable to levels reported for rural and urban schools built in the same era in the United States.^17,19,20^ These levels exceed Vermont’s screening value, and room 203 exceeds the action level for use by elementary school students (60 ng m^–3^). Although deployed only a few meters apart, the three samplers deployed in room 203 captured differences of more than 80 ng m^–3^ airborne PCB concentrations. The concentration captured by the sampler closest to the door (96 ng m^–3^) is lower than that of the sampler near the glass-block windows (180 ng m^–3^) (Figure 1). We hypothesize that this is due to poor mixing in the unventilated room and the presence of a strong emission source, but the small number of samples confounds confirmation.^26^

**Table 3:** Air Concentrations and Emissions of ΣPCBs Measured in Three Schoolrooms^a^

room	material	air concentration (ng m^–3^)	emissions (ng m^–2^ d^–1^)
201	carpet		300
201	carpet		280
201	carpet		270
203	air	180	
203	air	150	
203	air	96	
203	glass blocks		30,000
203	painted brick wall		2700
203	painted cinderblock wall		1700
203	tile		1400
203	painted drywall		1100
203	cove base		510
203	carpet		40
203	carpet		35
203	carpet		35
205	air	51	
205	air	41	
205	air	38	
205	painted cinderblock wall		560
205	painted drywall		480
205	carpet		410
205	carpet		360
205	carpet		220
Low-Volume Sampler Results (State of Vermont)			
203	air	120	
112	air	110	
112	air	95	
202	air	9.7	
201	air	ND (RL 58 ng m^–3^)	

a State of Vermont low-vol air results from select rooms are shown for comparison. ND indicates a non-detect and RL is the laboratory reporting limit.

Representatives of the State of Vermont collected air samples around the school using low-volume PUF sampling (U.S. EPA Method TO-10A).^30^ They analyzed 25 active samplers at this school, operated for 24 h, including two duplicates and one field blank. PCBs were identified by the consulting laboratory by Aroclor: 4 of 25 samples were identified as Aroclor 1254 and 3 exceeded Vermont’s action level (Table 3). One sample measured above detection limits but below Vermont’s action level and was identified as Aroclor 1254. PCBs were not detected in the remaining 21 samples, possibly due to the laboratory’s high detection limits (5.8–120 ng m^–3^). This variability between rooms in close proximity suggests that local sources are contributing to significantly different concentrations across classrooms. Room 203 air concentrations were consistent between active and passive samplers, indicating the comparability of the different sampling methods.

**Figure 3:**
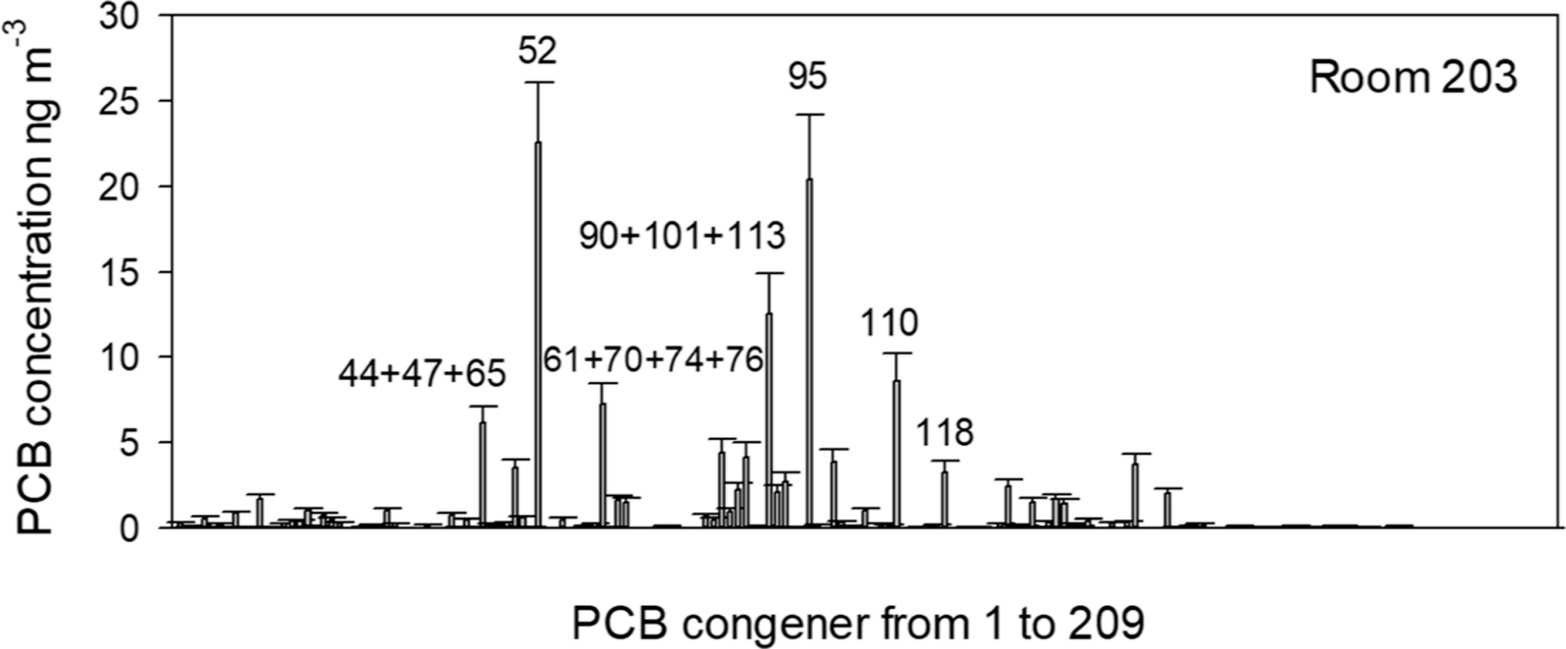
Average concentration profile of the airborne PCB congeners measured in room 203 (*n* = 3). Error bars represent the standard error.

The dominant congeners in Aroclor 1254 are also dominant in the air samples at this school (Figure 3). Aroclor 1254 is a well-studied mixture of PCBs associated with a wide range of adverse health outcomes.^31^ As a group of compounds, PCBs are known human carcinogens with most of the cancer-associated toxicity due to the dioxin-like compounds.^32^ PCB 118 (2,3′,4,4′,5-pentachlorobiphenyl) is a dioxin-like congener present in every sample with an average air concentration of 3 ng m^–3^. The top congeners in the school samples by mass are the nondioxin-like PCB 52 (2,2′,5,5′-tetrachlorobiphenyl) and PCB 95. PCB 52 and PCB 95, and the human metabolites of these compounds, have been identified as toxicants associated with neurological disorders like attention deficit and autism and metabolic disorders like obesity.^33–42^

We found emissions of PCBs from every sampled surface. Emissions varied drastically by the building material and location. Emissions from the glass-block windows (30,000 ng m^–2^ d^–1^) greatly exceeded those from all other surfaces, which ranged from 35 ng m^–2^ d^–1^ (carpet) to 2700 ng m^–2^ d^–1^ (brick wall). Emissions from the carpet in room 203 were greater than those in rooms 201 and 205, but overall, the carpet exhibited the lowest emissions of all materials. The tile sample was collected only 1 m apart from carpet samples in room 203, yet its emissions are 2 orders of magnitude greater and comparable to wall emissions. School officials reported that the tile and adhesive mastic were removed before the carpet was installed. Emissions off walls, except glass blocks, were similar within the room, regardless of material type. We found no air or emission samples with ΣPCB less than LOQ.

The emissions in room 203 exceed emissions reported for Lake Michigan, Green Bay, and the Hudson River.^43,44^ The emissions in this room are comparable to emissions from heavily contaminated bodies of water such as the New York Harbor (3000 ng m^–2^ d^–1^)^45^ and the Indiana Harbor and Ship Canal (7000 ng m^–2^ d^–1^)^43^ although not as high as the emission from New Bedford Harbor, the largest PCB Superfund site in the United States (1,200,000 ng m^–2^ d^–1^).^46^ However, because these emissions occur indoors, the airborne PCB concentrations in this school are much higher than those found in air directly over or near outdoor emissions.

PCB emission profiles were compared to reported profiles for Aroclors and found to be most similar to Aroclor 1254 using the cos θ measure of similarity.^47^ This method describes the similarity of profiles from 0 (no correlation) to 1 (complete correlation). The similarity with Aroclor 1254 ranged from 0.34 for the emissions from carpet and cove base to 0.83 for the emissions from the glass blocks. Emissions from carpet and cove base also exhibit similarity to Aroclor 1248, an Aroclor mixture that is more enriched in the lower chlorinated congeners than Aroclor 1254.

We are unaware of any prior report of the use of Aroclor in glass-block windows. Our measurements indicate that a reservoir of PCBs is present in the window materials, and we presume Aroclor was mixed in with the mortar and/or sealant used to install the blocks.^48^ We cannot be sure if Aroclors were used in other materials in the school rooms or if the emissions from the walls, carpet, and tile instead reflect secondary emissions due to diffusion or deposition of PCBs to those surfaces over many decades.

### Performance of the PUF-PES Device in Representing Aroclor Congener Emissions

The PCB congener profiles of emissions measured using the PUF-PES in this school are not exactly the same as any reported Aroclor mixture, although the magnitude of emissions from the glass-block windows strongly suggests the presence of a commercial mixture. Most apparent is the absence of the highest-molecular-weight congeners. We considered four explanations for this difference: (1) an unknown Aroclor mixture was used in the school; (2) weathering of the Aroclor over the decades since 1958 that changed the composition of PCBs that remain in the building materials; (3) variations in PCB congener volatility that cause differences in the rate of emissions; and (4) sampling artifacts in the accumulation of PCBs on the PUF-PES.

Interestingly, there is significant variability between different production lots of Aroclor 1254, and it is possible that the mixture installed in this school is not the same as has been reported in the literature.^49–52^

**Figure 4:**
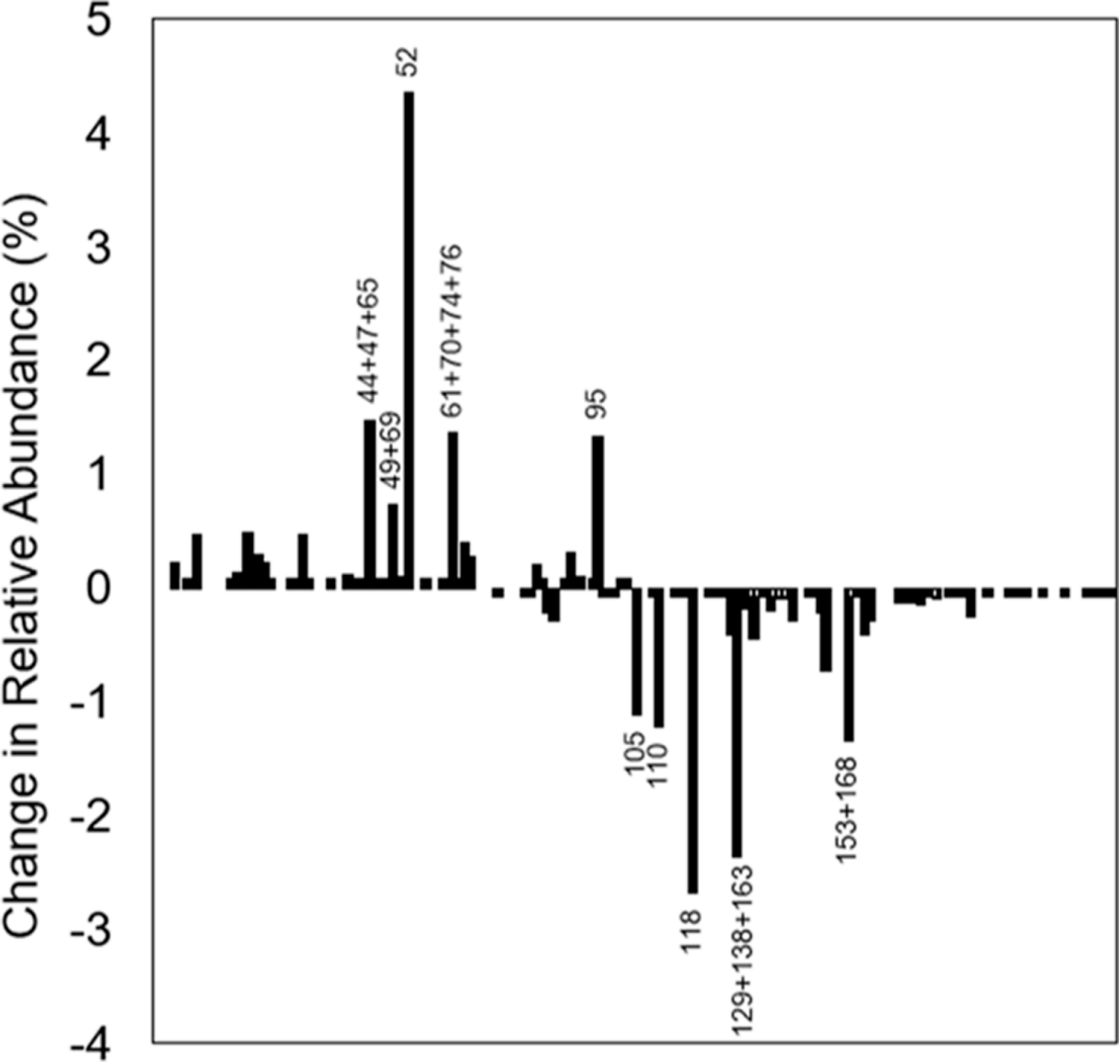
Percent difference between the emissions measured in this study and Aroclor 1254 (Rushneck, 2004), reported by congener. The emission rate is higher for the lower-molecular-mass congeners. This is due to the physical-chemical characteristics of the congeners, including diffusivity in the solid and the solid/air equilibrium concentration ratio. The congener order is from the lowest to the highest molecular mass, as listed in the Supporting Information. Ten congeners with the highest absolute differences are labeled.

To examine the effect of weathering due to photochemical reactions and volatilization, we considered the congeners that would be most subject to change. We know of no abiotic chemical reactions that would remove PCBs from solid materials, and we find no studies that show PCB breakdown in sunlight or heat. On the contrary, Aroclors were developed and marketed on the premise that this could not occur. Weathering due to volatilization is expected. The lower chlorinated congeners are more likely to volatilize and disperse over time, creating the potential for the Aroclor reservoir in the classroom to change to a mixture that is depleted in lower chlorinated congeners.^47^ However, the emissions showed no depletion of the lower chlorinated congeners. In fact, the lower chlorinated congeners are enriched in the emissions. The higher-molecular-weight congeners are underrepresented in the emissions (Figure 4). We conclude that the emissions do not support the conclusion that weathering changed the congener profile in an Aroclor since its installation in the classroom more than 50 years ago.

To examine the effect of congener volatility on the PCB profile, we normalized the Aroclor profile by the vapor pressure of each congener. We then compared this volatilized signature to the Aroclor congener distributions. We found an improvement in the similarity between most emissions and Aroclors. The volatility of the PCB congeners could affect both the release of PCBs from the solid surface and their uptake onto the PUF. While the differential release of congeners due to volatilization increases the similarity, a sampling artifact could form due to this effect as well. Such an artifact would cause a misrepresentation of the PCB congener emissions.

We examined whether a sampling artifact could explain the difference between the Aroclor and the emission congener signal. When emissions arise from a large reservoir of Aroclor, the net direction of flux is always from the Aroclor source to the air. Such a reservoir exists in schoolrooms when Aroclors were purposely added to adhesives, caulking, window glazing, or other fluid-like materials. In this situation, uptake on the PUF-PES is linear, and all the emissions are captured by the PUF. In this situation, our measurements of PCB emissions are not subject to any sampling artifacts due to equilibrium with the PUF. However, all congener emissions may be underestimated due to the stagnant conditions in the PUF-PES. Because the system prevents any turbulence from inducing higher emissions, the emissions could be higher under normal school conditions when children are present.

**Figure 5:**
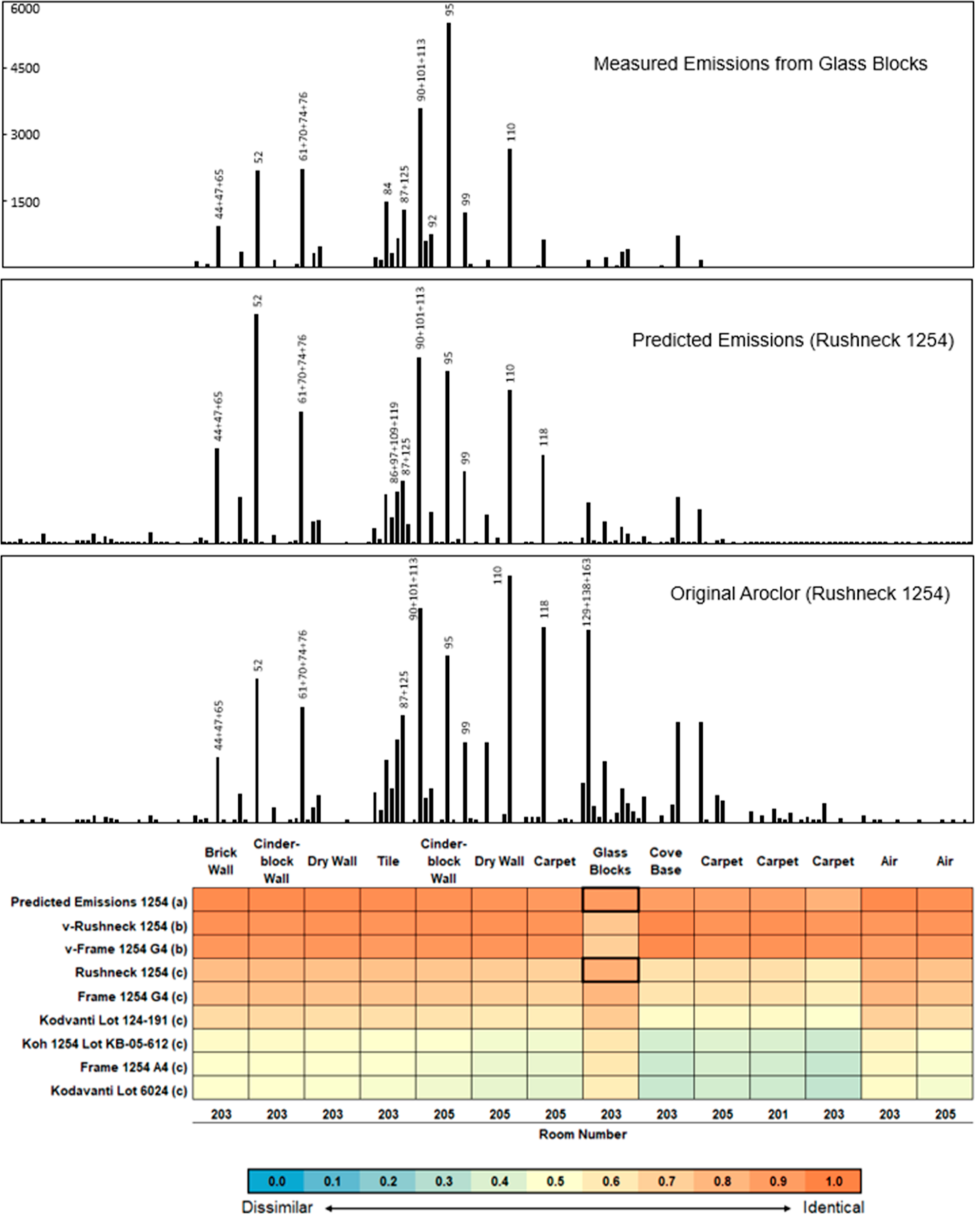
Predicted PCB congener emissions from Aroclor 1254 are highly correlated to measured emissions and air concentrations in the schoolrooms. For clarity, duplicate samples are not shown. The 10 highest congeners in each profile are labeled. Top panel: congener profiles for measured emissions from glass-block windows (ng m^–2^ d^–1^), the predicted congener profile for emissions of Aroclor 1254, and original Aroclor 1254. Bottom panel: the cosine theta similarity values for (a) our predicted congener distribution; (b) the volatilized form of three Aroclor 1254 lots; and (c) several production lots of Aroclor 1254 as reported in the literature. Cells in bold correspond to the profiles in the top panel.

To further probe the potential for differential congener emissions due to thermodynamic properties, we adapted a model simulating PCB flux from the surface to the air and the PUF. Details of the model are described elsewhere, and the parameters used are provided in the Supporting Information.^27^ Briefly, this model simulates mass release from a series of solid layers to air and uptake onto a series of PUF layers over time as a function of the solid and PUF characteristics. We used Rushneck’s Aroclor 1254 as the source present in the solid.^50,53,54^ The simulated mass of each congener emitted over 34 days was compared to the measured emissions, measured air concentrations, and reported distributions of Aroclor 1254 in the literature.^49–52^ We found the congener profile predicted to be emitted from a reservoir of Aroclor 1254 to be nearly the same as the congener profile we measured in both the glass-block emissions and air samples in these schoolrooms (Figure 5). The predicted emissions from Aroclor 1254 indicate differential release on a congener-specific basis from a source. The lower-molecular-weight congeners are enriched, and the higher-molecular-weight congeners are depressed in emissions.

Differences in congener signals are due to the diffusion of each congener from the solid to the air and not because of differential uptake in the PUF. The model’s results show that the PUF-PES is effective in capturing what is emitted from a source, and therefore, the difference seen between congener signals is not due to a sampling artifact of the PUF-PES. The strong similarity indicates that Aroclor 1254 remains present in the room, most likely in the mortar or sealant between the glass blocks. We concluded that the PUF-PES sample accurately represents the magnitude and distribution of PCB congener emissions released from surfaces. In the case of Aroclors purposely installed in building materials, depletion due to volatilization is an insignificant loss. Until physically removed, school building materials containing Aroclors will be a constant emission source to the room.

## Implications

The U.S. EPA’s implementation of the Toxics Substances Control Act requires removal of building materials containing 50 ppm of PCBs but is silent about materials that passively emit PCBs into indoor air. In Vermont, state regulations require action to reduce elevated airborne PCBs in schools and rely on EPA methods to identify schools and school rooms in need of remediation. However, the Aroclor-based methods for airborne PCBs do not identify or prioritize specific materials as emission sources.

This study provides a new method for prioritizing remediation and cost-effective reduction of children’s inhalation exposure to PCBs. Through direct measurement of emissions from classroom surfaces, we were able to identify the glass-block windows in this school as a major source. However, many questions remain about the presence of PCBs in school building materials. For example, we do not know how frequently Aroclors were used in the installation of glass-block windows, if Aroclors were premixed during manufacture into the products used in the installation, or if construction workers added Aroclor to the mortar or sealant when installing glass-block windows. Due to the tremendous uncertainty about the historical use of Aroclors in school building materials, direct measurements of air concentrations in individual schoolrooms and emissions from multiple building material surfaces are necessary to identify and reduce children’s exposure to PCBs.

## Supporting Information Available

Additional method details including extraction conditions; instrument settings; chemical structure identification; quality control data including analysis of standard reference material, surrogate recoveries, and congener-specific limits of quantification; effective sampling volume calculations; emission model parameters; Aroclor 1254 profiles; and cosine similarity analyses (PDF)
