# Supplementary material for: Polyurethane Foam Emission Samplers to Identify Sources of Airborne Polychlorinated Biphenyls from Glass-Block Windows and Other Room Surfaces in a Vermont School
Source: Environ Sci Technol. 2023 Sep 15;57(38):14310–8. doi: 10.1021/acs.est.3c05195 (PMC10537452; doi:10.1021/acs.est.3c05195)
Supplement: Supplementary file 1 — es3c05195_si_001.pdf [file es3c05195_si_001.pdf]

Supporting Information

Polyurethane Foam Emission Samplers to Identify Sources of Airborne Polychlorinated Biphenyls (PCBs) from Glass Block Windows and Other Room Surfaces in a Vermont School

Authors. Jason B.X. Hua, Rachel F. Marek, Keri C. Hornbuckle*

Department of Civil and Environmental Engineering, IIHR-Hydroscience & Engineering University of Iowa, Iowa City, Iowa USA 52242

*Corresponding author keri-hornbuckle@uiowa.edu Ph (319-384-0789)

Data generated in this research are available at https://doi.org/10.25820/data.006632[1].

Number of pages: 19

Number of tables: 6

Number of figures: 5

Method and Materials

PUF Extraction

We used accelerated solvent extraction with acetone and hexane (1:1 v/v) to extract PCBs from PUF samples (Tisch Environmental, Cleves, OH, Part # TE-1014). During ASE preparation, 9.84 ng of surrogate standard 13C labeled PCBs (**Table S1**) were added to the PUF to allow for corrections due to analytical losses and variability. Recoveries of 13C labeled surrogate standards were used to correct masses of corresponding homologs (**Table S1**). Turbulent evaporation with nitrogen (Biotage TurboVap II Automated Solvent Evaporation System) was used to concentrate extracts. Extracts were cleaned by sulfuric acid silica gel columns. Samples were then concentrated again, transferred to 2 mL glass autosampler vials, and spiked with 10.06 ng of internal standard d-PCB 30 (2,4,6-trichlorobiphenyl-2',3',4',5',6'-d5, C/D/N Isotopes) and internal standard PCB 204 (2,2',3,4,4',5,6,6'-octachlorobiphenyl, AccuStandard).

Table S1. Ten 13C labeled PCBs were used as surrogate standard. Each standard was used to correct masses of the corresponding homolog.

Surrogate Standard	Homolog
13 C 3	Monochlorinated
13 C 15	Dichlorinated
13 C 31	Trichlorinated
13 C 52	Tetrachlorinated
13 C 118	Pentachlorinated
13 C 153	Hexachlorinated
13 C 180	Heptachlorinated
13 C 194	Octachlorinated
13 C 206	Nonachlorinated
13 C 209	Decachlorinated

Instrument Parameters

The GC (Agilent 7000B Triple Quad with Agilent 7890A GC, Agilent 7693 autosampler, and multi-mode inlet) was equipped with a Supelco SPB-Octyl capillary column (Poly (50% n-octyl/50% methyl siloxane, 30 m × 0.25 mm ID, 0.25 µm film thicknesses)) with UHP helium as the carrier gas (constant flow 0.8 mL/min) and quench gas (2.25 mL/min) and UHP nitrogen as the collision gas (1.5 mL/min). The GC inlet operated at the following conditions: initial temperature 45 °C, initial time 0.06 min, ramp 600 °C/min to inlet temperature 325 °C. The GC oven temperature program was 45 °C for 2 min, 45 to 75 °C at 100 °C/min and hold for 5 min, 75 to 150 °C at 15 °C/min and hold for 1 min, 150 to 280 at 2.5 °C/min and final hold 6 min (total run time 71.3 min). Congener identity according to elution was assigned following the USEPA Method 1668C (**Table S2**). The MS transfer line temperature was held at 280 °C. The triple quadrupole MS electron ionization source was set to 260 °C, the dwell time 60 ms, and collision energy 25 V. The MS-MS operated with the precursor-product transitions in **Table S3**.

Table S2. Congener numbers and corresponding structures of all 209 PCBs in this study according to US EPA Method 1668C.

PCB#	Structure	PCB#	Structure	PCB#	Structure
1	2	44	2,2’,3,5’	87	2,2’,3,4,5’
2	3	45	2,2’,3,6	88	2,2’,3,4,6
3	4	46	2,2’,3,6’	89	2,2’,3,4,6’
4	2,2’	47	2,2’,4,4’	90	2,2’,3,4’,5
5	2,3	48	2,2’,4,5	91	2,2’,3,4’,6
6	2,3’	49	2,2’,4,5’	92	2,2’,3,5,5’
7	2,4	50	2,2’,4,6	93	2,2’,3,5,6
8	2,4’	51	2,2’,4,6’	94	2,2’,3,5,6’
9	2,5	52	2,2’,5,5’	95	2,2’,3,5’,6
10	2,6	53	2,2’,5,6’	96	2,2’,3,6,6’
11	3,3’	54	2,2’,6,6’	97	2,2’,3’,4,5
12	3,4	55	2,3,3’,4’	98	2,2’,3’,4,6
13	3,4’	56	2,3,3’,4’	99	2,2’,4,4’,5
14	3,5	57	2,3,3’,5	100	2,2’,4,4’,6
15	4,4’	58	2,3,3’,5’	101	2,2’,4,5,5’
16	2,2’,3	59	2,3,3’,6	102	2,2’,4,5,6’
17	2,2’,4	60	2,3,4,4’	103	2,2’,4,5’,6
18	2,2’,5	61	2,3,4,5	104	2,2’,4,6,6’
19	2,2’,6	62	2,3,4,6	105	2,3,3’,4,4’
20	2,3,3’	63	2,3,4’,5	106	2,3,3’,4,5
21	2,3,4	64	2,3,4’,6	107	2,3,3’,4’,5
22	2,3,4’	65	2,3,5,6	108	2,3,3’,4,5’
23	2,3,5	66	2,3’,4,4’	109	2,3,3’,4,6
24	2,3,6	67	2,3’,4,5	110	2,3,3’,4’,6
25	2,3’,4	68	2,3’,4,5’	111	2,3,3’,5,5’
26	2,3’,5	69	2,3’,4,6	112	2,3,3’,5,6
27	2,3’,6	70	2,3’,4’,5	113	2,3,3’,5’,6
28	2,4,4’	71	2,3’,4’,6	114	2,3,4,4’,5
29	2,4,5	72	2,3’,5,5’	115	2,3,4,4’,6
30	2,4,6	73	2,3’,5’,6	116	2,3,4,5,6
31	2,4’,5	74	2,4,4’,5	117	2,3,4’,5,6
32	2,4’,6	75	2,4,4’,6	118	2,3’,4,4’,5
33	2’,3,4	76	2’,3,4,5	119	2,3’,4,4’,6
34	2’,3,5	77	3,3’,4,4’	120	2,3’,4,5,5’
35	3,3’,4	78	3,3’,4,5	121	2,3’4,5’,6
36	3,3’,5	79	3,3’,4,5’	122	2’,3,3’,4,5
37	3,4,4’	80	3,3’,5,5’	123	2’,3,4,4’,5
38	3,4,5	81	3,4,4’,5	124	2’,3,4,5,5’
39	3,4’,5	82	2,2’,3,3’,4	125	2’,3,4,5,6’
40	2,2’,3,3’	83	2,2’,3,3’,5	126	3,3’,4,4’,5
41	2,2’,3,4	84	2,2’,3,3’,6	127	3,3’,4,5,5’
42	2,2’,3,4’	85	2,2’,3,4,4’	128	2,2’,3,3’,4,4’
43	2,2’,3,5	86	2,2’,3,4,5	129	2,2’,3,3’,4,5

Table S2 continued

PCB#	Structure	PCB#	Structure	
130	2,2’,3,3’,4,5’	171	2,2’,3,3’,4,4’,6	
131	2,2’,3,3’,4,6	172	2,2’,3,3’,4,5,5’	
132	2,2’,3,3’,4,6’	173	2,2’,3,3’,4,5,6	
133	2,2’,3,3’,5,5’	174	2,2’,3,3’,4,5,6’	
134	2,2’,3,3’,5,6	175	2,2’,3,3’,4,5’,6	
135	2,2’,3,3’,5,6’	176	2,2’,3,3’,4,6,6’	
136	2,2’,3,3’,6,6’	177	2,2’,3,3’,4’,5,6	
137	2,2’,3,4,4’,5	178	2,2’,3,3’,5,5’,6	
138	2,2’,3,4,4’,5’	179	2,2’,3,3’,5,6,6’	
139	2,2’,3,4,4’,6	180	2,2’,3,4,4’,5,5’	
140	2,2’,3,4,4’,6’	181	2,2’,3,4,4’,5,6	
141	2,2’,3,4,5,5’	182	2,2’,3,4,4’,5,6’	
142	2,2’,3,4,5,6	183	2,2’,3,4,4’,5’,6	
143	2,2’,3,4,5,6’	184	2,2’,3,4,4’,6,6’	
144	2,2’,3,4,5’,6	185	2,2’,3,4,5,5’,6	
145	2,2’,3,4,6,6’	186	2,2’,3,4,5,6,6’	
146	2,2’,3,4’,5,5’	187	2,2’,3,4’,5,5’,6	
147	2,2’,3,4’,5,6	188	2,2’,3,4’,5,6,6’	
148	2,2’,3,4’,5,6’	189	2,3,3’,4,4’,5,5’	
149	2,2’,3,4’,5’,6	190	2,3,3’,4,4’,5,6	
150	2,2’,3,4’,6,6’	191	2,3,3’,4,4’,5’,6	
151	2,2’,3,5,5’,6	192	2,3,3’,4,5,5’,6	
152	2,2’,3,5,6,6’	193	2,3,3’,4’,5,5’,6	
153	2,2’,4,4’,5,5’	194	2,2’,3,3’,4,4’,5,5’	
154	2,2’,4,4’,5’,6	195	2,2’,3,3’,4,4’,5,6	
155	2,2’,4,4’,6,6’	196	2,2’,3,3’,4,4’,5,6’	
156	2,3,3’,4,4’,5	197	2,2’,3,3’,4,4’,6,6’	
157	2,3,3’,4,4’,5’	198	2,2’,3,3’,4,5,5’,6	
158	2,3,3’,4,4’,6	199	2,2’,3,3’,4,5,5’,6’	
159	2,3,3’,4,5,5’	200	2,2’,3,3’,4,5,6,6’	
160	2,3,3’,4,5,6	201	2,2’,3,3’,4,5’,6,6’	
161	2,3,3’,4,5’,6	202	2,2’,3,3’,5,5’,6,6’	
162	2,3,3’,4’,5,5’	203	2,2’,3,4,4’,5,5’,6	
163	2,3,3’,4’,5,6	204	2,2’,3,4,4’,5,6,6’	
164	2,3,3’,4’,5’,6	205	2,3,3’,4,4’,5,5’,6	
165	2,3,3’,5,5’,6	206	2,2’,3,3’,4,4’,5,5’,6	
166	2,3,4,4’,5,6	207	2,2’,3,3’,4,4’,5,6,6’	
167	2,3’,4,4’,5,5’	208	2,2’,3,3’,4,5,5’,6,6’	
168	2,3’,4,4’,5’,6	209	2,2’,3,3’,4,4’,5,5’,6,6’	
169	3,3’,4,4’,5,5’			
170	2,2’,3,3’,4,4’,5			

Table S3. PCB precursor and product masses of unlabeled and deuterated calibration standards used in multiple reaction monitoring (MRM) mode on the triple quadrupole mass spectrometer[α].

Cl homolog	Precursor Mass	Product Mass
mono	188	153.1
di	222	152.1
tri	256	186
tetra	291.9	222
penta	325.9	255.9
hexa	359.8	289.9
hepta	393.8	323.9
octa	429.8	359.8
nona	463.7	393.8
deca	497.7	427.7
D5 tri	261	191.1
13C mono	200.1	165.1
13C di	234	164.1
13C tri	268	191.8
13C tetra	304	234
13C penta	337.9	268
13C hexa	371.9	301.9
13C hepta	405.8	335.9
13C octa	429.8	359.8
13C nona	475.8	405.8
13C deca	509.7	439.8

[α]Unlabeled standards were from AccuStandard, New Haven, CT, USA. Deuterated PCB 30 was from C/D/N Isotopes, Pointe-Claire, QC, Canada. 13C labeled standards were from Cambridge Isotope Laboratories, Tewksbury, MA, USA.

Quality Assurance and Quality Control

Table S4. Limit of quantification (LOQ) for each PCB congener or group of co-eluding congeners in unites of nanograms. [β]

PCB	LOQ	PCB	LOQ	PCB	LOQ	PCB	LOQ
1	0.52394	51	0.29012	107	0.39479	162	0.07787
2	0.34932	52	1.19083	108+124	0.40985	164	0.15887
3	1.15247	54	0.24473	110	0.93298	165	0.06292
4	0.66197	55	0.19713	111	0.09282	167	0.08465
5	0.53380	56	0.83213	112	0.09419	169	0.09970
6	0.46442	57	0.24541	114	0.11962	170	0.08842
7	0.30251	58	0.11372	115	0.20246	171+173	0.11190
8	0.81008	59+62+75	0.49225	117	0.30057	172	0.10962
9	0.32830	60	0.42554	118	0.71876	174	0.23119
10	0.21774	61+70 +74+76	0.87455	120	0.08632	175	0.09011
11	0.89008	63	0.12284	121	0.10286	176	0.19748
12+13	0.53342	64	0.78394	122	0.08100	177	0.12677
14	0.33225	66	0.90829	123	0.29451	178	0.21025
15	0.48058	67	0.10269	126	0.05292	179	0.35149
16	0.59609	68	0.14419	127	0.07463	180+193	0.08089
17	0.54228	72	0.16746	128+166	0.69030	181	0.12845
18+30	0.73809	73	0.14192	129+138 +163	0.69244	182	0.22438
19	0.32782	77	0.11967	130	0.10781	183	0.13832
20+28	0.91365	78	0.10771	131	0.08776	184	0.62014
21+33	0.78979	79	0.10628	132	1.12933	185	0.08250
22	0.66729	80	0.22318	133	0.11843	186	0.07928
23	0.22953	81	0.13374	134	0.32650	187	0.45474
24	0.16497	82	0.57477	135+151	0.77743	188	0.39803
25	0.36344	83	0.32591	136	0.45381	189	0.11946
26+29	0.46499	84	0.65204	137	0.20970	190	0.07405
27	0.27495	85+116	0.71888	139+140	0.21981	191	0.07876
31	0.88255	86+97+ 109+119	0.53748	141	0.62387	192	0.10497
32	0.49025	87+125	0.68189	142	0.14786	194	0.09782
34	0.29166	88	0.41198	143	0.16930	195	0.08408
35	0.10926	89	0.14504	144	0.54598	196	0.07941
36	0.07407	90+101 +113	0.98193	145	0.07422	197	0.09532
37	0.69488	91	0.69743	146	0.46312	198+199	0.08890

Table S4 continued

38	0.09472	92	0.54572	147+149	0.71895	200	0.06925
39	0.08271	93+100	0.26035	148	0.08805	201	0.07055
40+71	0.97483	94	0.17186	150	0.05471	202	0.08058
41	0.63036	95	1.13859	152	0.12865	203	0.07995
42	0.71217	96	0.41717	153+168	2.44711	205	0.07937
43	0.33684	98	0.19659	154	0.18046	206	0.12587
44+47+65	3.03149	99	0.61899	155	0.09442	207	0.12813
45	0.51265	102	0.38398	156+157	0.18337	208	0.15718
46	0.48403	103	0.26219	158	0.40305	209	0.59358
48	0.83899	104	0.06649	159	0.09300		
49+69	0.68390	105	0.51607	160	0.12949		
50+53	0.45048	106	0.12313	161	0.06932		

[β]The LOQ was calculated as the upper limit of the 99% confidence interval of the log 10 transformed mass in the blanks (average + 2.325 * standard deviation).

Standard Reference Material

We assessed the quality of our method using a standard reference material (SRM, 2585, National Institutes of Standards and Technology). Our results were compared to the certified concentrations reported by NIST. Congeners in parenthesis indicate that our analytical method include other congeners that coelute with the certified congener. Certified results only measure the first listed congener.

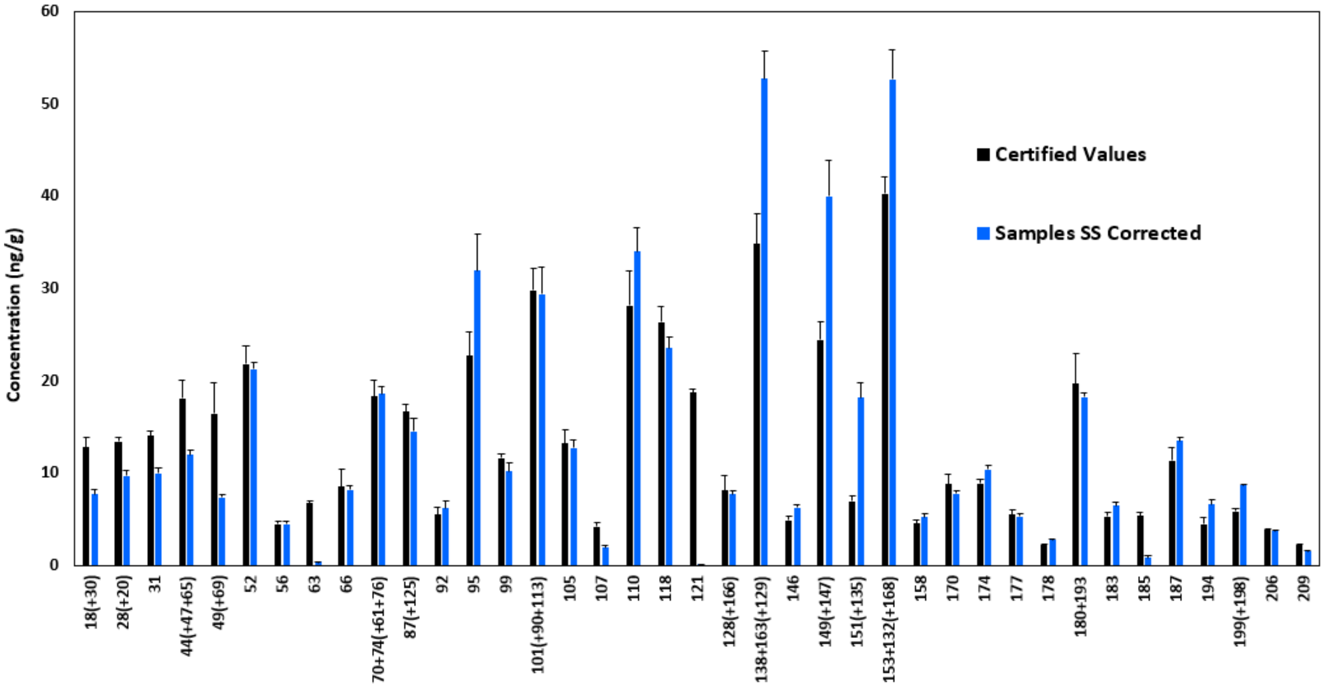

Figure S1. Analysis of National Institutes of Standards and Technology Standard Reference Material (SRM) 2585, organic contaminants in house dust (n = 4).

Table S5. Mean surrogate standard recoveries as percentages by congener.

PCB	Mean Recovery (%)	Standard Deviation (%)
13C 3	66	12
13C 15	78	9
13C 31	80	9
13C 52	84	10
13C 118	84	10
13C 153	85	10
13C 180	84	9
13C 194	82	9
13C 206	83	10
13C 209	84	9

Determining Sample Effective Volume

The variable K_{PUF} is calculated by the empirical equation (Shoeib[2]):

$$\log K_{\mathrm{PUF}} = 0.6366 \log K_{OA} - 3.1774 \qquad (eq\ S1)$$

where K_{OA} is calculated by (Herkert[3]):

$$\log K_{\mathrm{OA(T)}} = \log K_{OA(25°\mathrm{C})} - \frac{\Delta U_{OA}}{2.303 * R}\left(\frac{1}{T} - \frac{1}{298.15}\right) \qquad (eq\ S2)$$

where T is the temperature (K), ΔU_{OA} is the internal energy of octanol-air transfer (J mol^{-1}), R is the gas constant (J mol^{-1} K^{-1}).

The sampling rate, R_s, is congener specific and calculated by:

$$R_{\mathrm{s}} = \left(f_{\mathrm{on}}\sqrt{WS_{\mathrm{on}}} + f_{\mathrm{off}}\sqrt{WS_{\mathrm{off}}}\right)\left(\frac{1}{\sqrt[3]{\mathrm{MW}}}\right)10^{(0.0012T+c)} \qquad (eq\ S3)$$

where f_{on} and f_{off} are the fraction of the day the ventilation is on/off (unitless), WS_{on} and WS_{off} is the wind speed (m s^{-1}) when the ventilation is on/off, MW is the molecular weight (g mol^{-1}), T is the room temperature (°C), and c is an empirical sampler constant (unitless). The effective volume, V_{eff}, is then calculated as:

$$V_{\mathrm{eff}} = (V_{\mathrm{PUF}}K_{\mathrm{PUF}})\left[1 - e^{-\left(\frac{R_s}{V_{\mathrm{PUF}}K_{\mathrm{PUF}}}\right)t}\right] \qquad (eq\ S4)$$

where V_{PUF} is the PUF volume (m^3) and t is the deployment time (days). The final concentration in air, C_{air} is:

$$C_{air} = \sum\frac{M_{PCBi}}{V_{eff}} \qquad (eq\ S5)$$

where M_{PCBi} is the mass of a PCB (ng).

Emissions, E, are calculated as:

$$E = \sum\frac{M_{PCBi}}{At} \qquad (eq\ S6)$$

where A is the area of the PUF-PES (m^2) and t is the deployment time (days).

Modeling

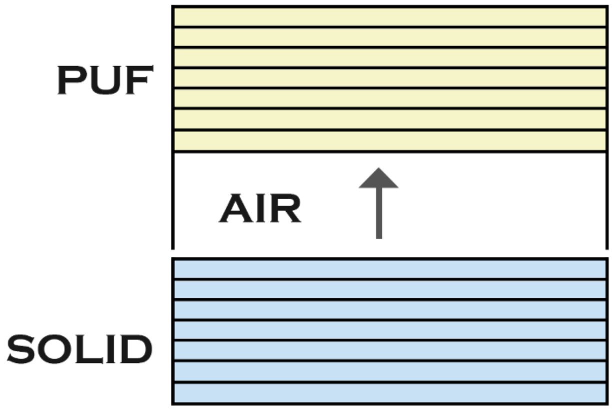

Figure S2. Representation of a diffusional, solid source emitting PCBs into the PUF-PES.

Our model was adapted from Jahnke *et al.* (2019).[4] We assumed the solid surface is represented by ten slices. The top slice is exposed to the air, releasing PCBs into the PUF-PES. The PUF-PES encases the surface, preventing turbulence within this control volume. Therefore, diffusion is the main driver of PCB flux.

Table S6. In addition to constants used in Eq. S1-4, congener specific parameters used in the model include kma, the mass transfer coefficient (unitless); Koa, the octanol-air partitioning coefficient (unitless); Dm, solid-phase diffusion coefficient for the solid source ($m^2\ h^{-1}$); DmPUF, the solid-phase diffusion coefficient for the PUF sink ($m^2\ h^{-1}$); kaPES, the gas phase mass transfer coefficient for the source ($m\ h^{-1}$).

PCB	k_{ma}	K_{oa}	K_{PUF}	MW	D_m	D_{mPUF}	k_{aPES}
1	8.17x10^5	7.00x10^6	1.23x10^5	188.65	2.12x10^-10	7.22x10^-10	4.33
2	8.17x10^5	7.00x10^6	1.74x10^5	188.65	2.12x10^-10	7.22x10^-10	4.33
3	8.17x10^5	7.00x10^6	1.79x10^5	188.65	2.12x10^-10	7.22x10^-10	4.33
4	6.57x10^5	8.64x10^6	1.51x10^5	223.1	1.40x10^-10	4.76x10^-10	3.98
5	1.24x10^6	1.37x10^7	2.93x10^5	223.1	1.40x10^-10	4.76x10^-10	3.98
6	1.15x10^6	1.22x10^7	2.74x10^5	223.1	1.40x10^-10	4.76x10^-10	3.98
7	1.07x10^6	1.08x10^7	2.54x10^5	223.1	1.40x10^-10	4.76x10^-10	3.98
8	1.26x10^6	1.41x10^7	2.91x10^5	223.1	1.40x10^-10	4.76x10^-10	3.98
9	1.03x10^6	1.02x10^7	2.76x10^5	223.1	1.40x10^-10	4.76x10^-10	3.98
10	6.82x10^5	9.06x10^6	1.70x10^5	223.1	1.40x10^-10	4.76x10^-10	3.98
11	1.25x10^6	1.39x10^7	4.03x10^5	223.1	1.40x10^-10	4.76x10^-10	3.98
12	2.29x10^6	3.70x10^7	4.23x10^5	223.1	1.40x10^-10	4.76x10^-10	3.98
13	2.25x10^6	3.59x10^7	4.45x10^5	223.1	1.40x10^-10	4.76x10^-10	3.98
14	2.25x10^6	3.59x10^7	3.18x10^5	223.1	1.40x10^-10	4.76x10^-10	3.98
15	2.21x10^6	3.49x10^7	4.89x10^5	223.1	1.40x10^-10	4.76x10^-10	3.98
16	2.50x10^6	4.87x10^7	3.73x10^5	257.54	9.78x10^-11	3.33x10^-10	3.71
17	2.21x10^6	4.14x10^7	3.30x10^5	257.54	9.78x10^-11	3.33x10^-10	3.71
18	2.05x10^6	3.77x10^7	3.59x10^5	257.54	9.78x10^-11	3.33x10^-10	3.71

Table S6 continued

19	1.40x10^6	2.31x10^7	2.14x10^5	257.54	9.78x10^-11	3.33x10^-10	3.71
20	4.63x10^6	1.15x10^8	7.37x10^5	257.54	9.78x10^-11	3.33x10^-10	3.71
21	4.72x10^6	1.19x10^8	6.56x10^5	257.54	9.78x10^-11	3.33x10^-10	3.71
22	5.16x10^6	1.38x10^8	8.11x10^5	257.54	9.78x10^-11	3.33x10^-10	3.71
23	3.41x10^6	7.03x10^7	5.77x10^5	257.54	9.78x10^-11	3.33x10^-10	3.71
24	3.89x10^5	4.39x10^6	4.25x10^5	257.54	9.78x10^-11	3.33x10^-10	3.71
25	3.94x10^6	8.88x10^7	6.72x10^5	257.54	9.78x10^-11	3.33x10^-10	3.71
26	3.80x10^6	8.37x10^7	6.88x10^5	257.54	9.78x10^-11	3.33x10^-10	3.71
27	2.37x10^6	4.54x10^7	4.15x10^5	257.54	9.78x10^-11	3.33x10^-10	3.71
28	4.39x10^6	1.06x10^8	7.16x10^5	257.54	9.78x10^-11	3.33x10^-10	3.71
29	3.00x10^6	5.73x10^7	6.32x10^5	257.54	9.78x10^-11	3.33x10^-10	3.71
30	2.05x10^6	3.77x10^7	3.32x10^5	257.54	9.78x10^-11	3.33x10^-10	3.71
31	4.15x10^6	9.69x10^7	7.36x10^5	257.54	9.78x10^-11	3.33x10^-10	3.71
32	2.74x10^6	5.48x10^7	4.23x10^5	257.54	9.78x10^-11	3.33x10^-10	3.71
33	4.72x10^6	1.19x10^8	7.23x10^5	257.54	9.78x10^-11	3.33x10^-10	3.71
34	3.41x10^6	7.03x10^7	5.51x10^5	257.54	9.78x10^-11	3.33x10^-10	3.71
35	9.04x10^6	3.40x10^8	6.88x10^5	257.54	9.78x10^-11	3.33x10^-10	3.71
36	6.65x10^6	2.07x10^8	8.68x10^5	257.54	9.78x10^-11	3.33x10^-10	3.71
37	9.89x10^6	3.93x10^8	1.34x10^6	257.54	9.78x10^-11	3.33x10^-10	3.71
38	9.72x10^6	3.82x10^8	9.22x10^5	257.54	9.78x10^-11	3.33x10^-10	3.71
39	9.72x10^6	3.82x10^8	9.49x10^5	257.54	9.78x10^-11	3.33x10^-10	3.71
40	9.54x10^6	2.75x10^8	9.32x10^5	291.99	7.16x10^-11	2.43x10^-10	3.48
41	9.54x10^6	2.75x10^8	8.10x10^5	291.99	7.16x10^-11	2.43x10^-10	3.48
42	8.72x10^6	2.44x10^8	8.71x10^5	291.99	7.16x10^-11	2.43x10^-10	3.48
43	6.65x10^6	1.72x10^8	7.23x10^5	291.99	7.16x10^-11	2.43x10^-10	3.48
44	7.96x10^6	2.17x10^8	9.19x10^5	291.99	7.16x10^-11	2.43x10^-10	3.48
45	4.72x10^6	1.10x10^8	5.11x10^5	291.99	7.16x10^-11	2.43x10^-10	3.48
46	7.34x10^6	1.96x10^8	5.26x10^5	291.99	7.16x10^-11	2.43x10^-10	3.48
47	7.54x10^6	2.03x10^8	7.89x10^5	291.99	7.16x10^-11	2.43x10^-10	3.48
48	7.28x10^6	1.93x10^8	8.06x10^5	291.99	7.16x10^-11	2.43x10^-10	3.48
49	6.89x10^6	1.80x10^8	8.10x10^5	291.99	7.16x10^-11	2.43x10^-10	3.48
50	5.96x10^6	1.50x10^8	4.36x10^5	291.99	7.16x10^-11	2.43x10^-10	3.48
51	5.16x10^6	1.24x10^8	4.69x10^5	291.99	7.16x10^-11	2.43x10^-10	3.48
52	6.41x10^6	1.64x10^8	8.51x10^5	291.99	7.16x10^-11	2.43x10^-10	3.48
53	4.15x10^6	9.37x10^7	5.16x10^5	291.99	7.16x10^-11	2.43x10^-10	3.48
54	9.12x10^6	2.59x10^8	2.38x10^5	291.99	7.16x10^-11	2.43x10^-10	3.48
55	9.12x10^6	3.45x10^8	1.78x10^6	291.99	7.16x10^-11	2.43x10^-10	3.48
56	2.00x10^7	1.23x10^9	2.01x10^6	291.99	7.16x10^-11	2.43x10^-10	3.48
57	2.00x10^7	1.23x10^9	1.52x10^6	291.99	7.16x10^-11	2.43x10^-10	3.48
58	2.00x10^7	1.23x10^9	1.59x10^6	291.99	7.16x10^-11	2.43x10^-10	3.48
59	2.04x10^7	7.33x10^8	1.03x10^6	291.99	7.16x10^-11	2.43x10^-10	3.48
60	2.04x10^7	1.27x10^9	2.00x10^6	291.99	7.16x10^-11	2.43x10^-10	3.48

Table S6 continued

61	1.70x10^7	9.45x10^8	1.36x10^6	291.99	7.16x10^-11	2.43x10^-10	3.48
62	1.70x10^7	5.80x10^8	8.34x10^5	291.99	7.16x10^-11	2.43x10^-10	3.48
63	1.42x10^7	7.06x10^8	1.63x10^6	291.99	7.16x10^-11	2.43x10^-10	3.48
64	8.41x10^6	2.33x10^8	1.11x10^6	291.99	7.16x10^-11	2.43x10^-10	3.48
65	7.96x10^6	2.17x10^8	8.48x10^5	291.99	7.16x10^-11	2.43x10^-10	3.48
66	1.67x10^7	9.18x10^8	1.90x10^6	291.99	7.16x10^-11	2.43x10^-10	3.48
67	1.45x10^7	7.26x10^8	1.74x10^6	291.99	7.16x10^-11	2.43x10^-10	3.48
68	9.80x10^6	3.88x10^8	1.40x10^6	291.99	7.16x10^-11	2.43x10^-10	3.48
69	6.65x10^6	1.72x10^8	8.51x10^5	291.99	7.16x10^-11	2.43x10^-10	3.48
70	1.58x10^7	8.41x10^8	1.91x10^6	291.99	7.16x10^-11	2.43x10^-10	3.48
71	9.89x10^6	2.88x10^8	1.06x10^6	291.99	7.16x10^-11	2.43x10^-10	3.48
72	1.25x10^7	5.75x10^8	1.44x10^6	291.99	7.16x10^-11	2.43x10^-10	3.48
73	1.41x10^7	4.54x10^8	8.36x10^5	291.99	7.16x10^-11	2.43x10^-10	3.48
74	1.58x10^7	8.41x10^8	1.84x10^6	291.99	7.16x10^-11	2.43x10^-10	3.48
75	1.57x10^7	5.22x10^8	9.12x10^5	291.99	7.16x10^-11	2.43x10^-10	3.48
76	1.55x10^7	8.16x10^8	1.55x10^6	291.99	7.16x10^-11	2.43x10^-10	3.48
77	4.05x10^7	3.84x10^9	3.67x10^6	291.99	7.16x10^-11	2.43x10^-10	3.48
78	2.70x10^7	1.99x10^9	2.69x10^6	291.99	7.16x10^-11	2.43x10^-10	3.48
79	2.20x10^7	1.43x10^9	2.64x10^6	291.99	7.16x10^-11	2.43x10^-10	3.48
80	1.80x10^7	1.03x10^9	1.92x10^6	291.99	7.16x10^-11	2.43x10^-10	3.48
81	2.49x10^7	1.74x10^9	2.97x10^6	291.99	7.16x10^-11	2.43x10^-10	3.48
82	2.93x10^7	1.17x10^9	2.33x10^6	326.43	5.42x10^-11	1.84x10^-10	3.29
83	3.44x10^7	1.44x10^9	2.01x10^6	326.43	5.42x10^-11	1.84x10^-10	3.29
84	1.76x10^7	6.08x10^8	1.32x10^6	326.43	5.42x10^-11	1.84x10^-10	3.29
85	2.27x10^7	8.43x10^8	2.27x10^6	326.43	5.42x10^-11	1.84x10^-10	3.29
86	2.93x10^7	1.17x10^9	1.78x10^6	326.43	5.42x10^-11	1.84x10^-10	3.29
87	3.03x10^7	1.23x10^9	2.30x10^6	326.43	5.42x10^-11	1.84x10^-10	3.29
88	1.55x10^7	5.16x10^8	1.05x10^6	326.43	5.42x10^-11	1.84x10^-10	3.29
89	1.98x10^7	7.08x10^8	1.24x10^6	326.43	5.42x10^-11	1.84x10^-10	3.29
90	2.53x10^7	9.70x10^8	1.89x10^6	326.43	5.42x10^-11	1.84x10^-10	3.29
91	1.67x10^7	5.67x10^8	1.23x10^6	326.43	5.42x10^-11	1.84x10^-10	3.29
92	2.08x10^7	7.50x10^8	2.01x10^6	326.43	5.42x10^-11	1.84x10^-10	3.29
93	1.45x10^7	4.70x10^8	1.09x10^6	326.43	5.42x10^-11	1.84x10^-10	3.29
94	1.42x10^7	4.59x10^8	1.14x10^6	326.43	5.42x10^-11	1.84x10^-10	3.29
95	1.39x10^7	4.49x10^8	1.35x10^6	326.43	5.42x10^-11	1.84x10^-10	3.29
96	2.02x10^7	7.24x10^8	6.03x10^5	326.43	5.42x10^-11	1.84x10^-10	3.29
97	2.93x10^7	1.17x10^9	2.33x10^6	326.43	5.42x10^-11	1.84x10^-10	3.29
98	1.47x10^7	4.81x10^8	1.14x10^6	326.43	5.42x10^-11	1.84x10^-10	3.29
99	2.53x10^7	9.70x10^8	2.19x10^6	326.43	5.42x10^-11	1.84x10^-10	3.29
100	2.40x10^7	9.05x10^8	1.04x10^6	326.43	5.42x10^-11	1.84x10^-10	3.29
101	2.27x10^7	8.43x10^8	2.18x10^6	326.43	5.42x10^-11	1.84x10^-10	3.29
102	1.79x10^7	6.19x10^8	1.23x10^6	326.43	5.42x10^-11	1.84x10^-10	3.29

Table S6 continued

103	1.41x10^7	4.54x10^8	1.10x10^6	326.43	5.42x10^-11	1.84x10^-10	3.29
104	3.40x10^7	1.42x10^9	4.79x10^5	326.43	5.42x10^-11	1.84x10^-10	3.29
105	8.20x10^7	1.20x10^10	5.70x10^6	326.43	5.42x10^-11	1.84x10^-10	3.29
106	6.66x10^7	8.56x10^9	4.07x10^6	326.43	5.42x10^-11	1.84x10^-10	3.29
107	5.41x10^7	6.12x10^9	4.61x10^6	326.43	5.42x10^-11	1.84x10^-10	3.29
108	5.76x10^7	2.81x10^9	4.33x10^6	326.43	5.42x10^-11	1.84x10^-10	3.29
109	4.54x10^7	4.60x10^9	2.31x10^6	326.43	5.42x10^-11	1.84x10^-10	3.29
110	3.57x10^7	1.51x10^9	2.91x10^6	326.43	5.42x10^-11	1.84x10^-10	3.29
111	3.12x10^7	2.51x10^9	3.57x10^6	326.43	5.42x10^-11	1.84x10^-10	3.29
112	2.72x10^7	1.07x10^9	2.29x10^6	326.43	5.42x10^-11	1.84x10^-10	3.29
113	3.38x10^7	1.41x10^9	2.30x10^6	326.43	5.42x10^-11	1.84x10^-10	3.29
114	7.16x10^7	9.62x10^9	4.52x10^6	326.43	5.42x10^-11	1.84x10^-10	3.29
115	3.57x10^7	1.51x10^9	2.56x10^6	326.43	5.42x10^-11	1.84x10^-10	3.29
116	3.38x10^7	1.41x10^9	1.80x10^6	326.43	5.42x10^-11	1.84x10^-10	3.29
117	3.20x10^7	1.31x10^9	2.48x10^6	326.43	5.42x10^-11	1.84x10^-10	3.29
118	6.14x10^7	7.51x10^9	5.60x10^6	326.43	5.42x10^-11	1.84x10^-10	3.29
119	2.93x10^7	1.17x10^9	2.52x10^6	326.43	5.42x10^-11	1.84x10^-10	3.29
120	2.36x10^7	1.60x10^9	3.93x10^6	326.43	5.42x10^-11	1.84x10^-10	3.29
121	1.90x10^7	6.68x10^8	1.89x10^6	326.43	5.42x10^-11	1.84x10^-10	3.29
122	3.44x10^7	2.95x10^9	4.84x10^6	326.43	5.42x10^-11	1.84x10^-10	3.29
123	6.25x10^7	7.73x10^9	4.55x10^6	326.43	5.42x10^-11	1.84x10^-10	3.29
124	4.94x10^7	5.29x10^9	4.54x10^6	326.43	5.42x10^-11	1.84x10^-10	3.29
125	3.91x10^7	1.70x10^9	2.38x10^6	326.43	5.42x10^-11	1.84x10^-10	3.29
126	1.42x10^8	2.92x10^10	9.06x10^6	326.43	5.42x10^-11	1.84x10^-10	3.29
127	1.51x10^8	3.21x10^10	6.56x10^6	326.43	5.42x10^-11	1.84x10^-10	3.29
128	1.60x10^8	1.05x10^10	6.28x10^6	360.88	4.22x10^-11	1.44x10^-10	3.13
129	1.22x10^8	7.41x10^9	5.12x10^6	360.88	4.22x10^-11	1.44x10^-10	3.13
130	1.04x10^8	6.01x10^9	5.37x10^6	360.88	4.22x10^-11	1.44x10^-10	3.13
131	7.10x10^7	3.68x10^9	2.93x10^6	360.88	4.22x10^-11	1.44x10^-10	3.13
132	6.72x10^7	3.43x10^9	3.28x10^6	360.88	4.22x10^-11	1.44x10^-10	3.13
133	6.03x10^7	2.98x10^9	4.88x10^6	360.88	4.22x10^-11	1.44x10^-10	3.13
134	5.41x10^7	2.59x10^9	2.90x10^6	360.88	4.22x10^-11	1.44x10^-10	3.13
135	4.20x10^7	1.87x10^9	3.00x10^6	360.88	4.22x10^-11	1.44x10^-10	3.13
136	2.63x10^7	1.02x10^9	1.46x10^6	360.88	4.22x10^-11	1.44x10^-10	3.13
137	1.72x10^8	1.16x10^10	4.95x10^6	360.88	4.22x10^-11	1.44x10^-10	3.13
138	1.18x10^8	7.07x10^9	6.21x10^6	360.88	4.22x10^-11	1.44x10^-10	3.13
139	5.61x10^7	2.71x10^9	2.75x10^6	360.88	4.22x10^-11	1.44x10^-10	3.13
140	7.29x10^7	3.81x10^9	2.87x10^6	360.88	4.22x10^-11	1.44x10^-10	3.13
141	9.48x10^7	5.34x10^9	5.09x10^6	360.88	4.22x10^-11	1.44x10^-10	3.13
142	7.10x10^7	3.68x10^9	2.24x10^6	360.88	4.22x10^-11	1.44x10^-10	3.13
143	5.31x10^7	2.53x10^9	2.66x10^6	360.88	4.22x10^-11	1.44x10^-10	3.13
144	6.48x10^7	3.27x10^9	2.94x10^6	360.88	4.22x10^-11	1.44x10^-10	3.13

Table S6 continued

145	2.77x10^7	1.09x10^9	1.20x10^6	360.88	4.22x10^-11	1.44x10^-10	3.13
146	7.63x10^7	4.04x10^9	5.37x10^6	360.88	4.22x10^-11	1.44x10^-10	3.13
147	6.14x10^7	3.05x10^9	2.70x10^6	360.88	4.22x10^-11	1.44x10^-10	3.13
148	5.51x10^7	2.65x10^9	2.54x10^6	360.88	4.22x10^-11	1.44x10^-10	3.13
149	4.94x10^7	2.30x10^9	3.39x10^6	360.88	4.22x10^-11	1.44x10^-10	3.13
150	4.48x10^7	2.03x10^9	1.27x10^6	360.88	4.22x10^-11	1.44x10^-10	3.13
151	4.05x10^7	1.78x10^9	2.90x10^6	360.88	4.22x10^-11	1.44x10^-10	3.13
152	5.92x10^7	2.91x10^9	1.22x10^6	360.88	4.22x10^-11	1.44x10^-10	3.13
153	8.66x10^7	4.75x10^9	6.09x10^6	360.88	4.22x10^-11	1.44x10^-10	3.13
154	4.20x10^7	1.87x10^9	2.85x10^6	360.88	4.22x10^-11	1.44x10^-10	3.13
155	1.09x10^8	6.37x10^9	1.05x10^6	360.88	4.22x10^-11	1.44x10^-10	3.13
156	2.80x10^8	8.72x10^10	1.31x10^7	360.88	4.22x10^-11	1.44x10^-10	3.13
157	2.85x10^8	8.98x10^10	1.35x10^7	360.88	4.22x10^-11	1.44x10^-10	3.13
158	1.24x10^8	7.59x10^9	7.04x10^6	360.88	4.22x10^-11	1.44x10^-10	3.13
159	1.18x10^8	2.15x10^10	9.99x10^6	360.88	4.22x10^-11	1.44x10^-10	3.13
160	1.15x10^8	6.83x10^9	5.24x10^6	360.88	4.22x10^-11	1.44x10^-10	3.13
161	1.13x10^8	6.71x10^9	5.43x10^6	360.88	4.22x10^-11	1.44x10^-10	3.13
162	1.12x10^8	1.99x10^10	1.11x10^7	360.88	4.22x10^-11	1.44x10^-10	3.13
163	1.11x10^8	6.59x10^9	6.73x10^6	360.88	4.22x10^-11	1.44x10^-10	3.13
164	1.53x10^8	9.93x10^9	6.76x10^6	360.88	4.22x10^-11	1.44x10^-10	3.13
165	1.79x10^8	1.22x10^10	5.32x10^6	360.88	4.22x10^-11	1.44x10^-10	3.13
166	1.94x10^8	1.35x10^10	5.76x10^6	360.88	4.22x10^-11	1.44x10^-10	3.13
167	2.10x10^8	5.47x10^10	1.30x10^7	360.88	4.22x10^-11	1.44x10^-10	3.13
168	3.42x10^8	2.81x10^10	5.80x10^6	360.88	4.22x10^-11	1.44x10^-10	3.13
169	5.57x10^8	2.65x10^11	2.24x10^7	360.88	4.22x10^-11	1.44x10^-10	3.13
170	5.00x10^8	4.59x10^10	1.50x10^7	395.32	3.37x10^-11	1.14x10^-10	2.99
171	2.30x10^8	1.68x10^10	7.77x10^6	395.32	3.37x10^-11	1.14x10^-10	2.99
172	3.12x10^8	2.50x10^10	1.34x10^7	395.32	3.37x10^-11	1.14x10^-10	2.99
173	2.40x10^8	1.78x10^10	6.57x10^6	395.32	3.37x10^-11	1.14x10^-10	2.99
174	1.85x10^8	1.27x10^10	7.87x10^6	395.32	3.37x10^-11	1.14x10^-10	2.99
175	1.39x10^8	8.73x10^9	7.20x10^6	395.32	3.37x10^-11	1.14x10^-10	2.99
176	8.66x10^7	4.75x10^9	3.33x10^6	395.32	3.37x10^-11	1.14x10^-10	2.99
177	1.99x10^8	1.39x10^10	7.70x10^6	395.32	3.37x10^-11	1.14x10^-10	2.99
178	1.24x10^8	7.59x10^9	7.09x10^6	395.32	3.37x10^-11	1.14x10^-10	2.99
179	7.49x10^7	3.94x10^9	3.34x10^6	395.32	3.37x10^-11	1.14x10^-10	2.99
180	3.55x10^8	2.94x10^10	1.50x10^7	395.32	3.37x10^-11	1.14x10^-10	2.99
181	2.28x10^8	1.66x10^10	6.47x10^6	395.32	3.37x10^-11	1.14x10^-10	2.99
182	1.46x10^8	9.36x10^9	6.88x10^6	395.32	3.37x10^-11	1.14x10^-10	2.99
183	1.63x10^8	1.08x10^10	8.03x10^6	395.32	3.37x10^-11	1.14x10^-10	2.99
184	1.69x10^8	1.13x10^10	2.88x10^6	395.32	3.37x10^-11	1.14x10^-10	2.99
185	1.75x10^8	1.18x10^10	6.77x10^6	395.32	3.37x10^-11	1.14x10^-10	2.99
186	1.60x10^8	1.05x10^10	2.62x10^6	395.32	3.37x10^-11	1.14x10^-10	2.99

Table S6 continued

187	1.46x10^8	9.36x10^9	7.65x10^6	395.32	3.37x10^-11	1.14x10^-10	2.99
188	3.66x10^8	3.07x10^10	2.82x10^6	395.32	3.37x10^-11	1.14x10^-10	2.99
189	9.16x10^8	5.91x10^11	7.75x10^6	395.32	3.37x10^-11	1.14x10^-10	2.99
190	5.37x10^8	5.04x10^10	1.69x10^7	395.32	3.37x10^-11	1.14x10^-10	2.99
191	3.95x10^8	3.39x10^10	1.73x10^7	395.32	3.37x10^-11	1.14x10^-10	2.99
192	3.12x10^8	2.50x10^10	1.31x10^7	395.32	3.37x10^-11	1.14x10^-10	2.99
193	3.55x10^8	2.94x10^10	1.64x10^7	395.32	3.37x10^-11	1.14x10^-10	2.99
194	1.71x10^9	2.25x10^11	3.79x10^7	429.77	2.73x10^-11	9.29x10^-11	2.87
195	9.08x10^8	9.92x10^10	1.87x10^7	429.77	2.73x10^-11	9.29x10^-11	2.87
196	6.21x10^8	6.07x10^10	2.01x10^7	429.77	2.73x10^-11	9.29x10^-11	2.87
197	3.39x10^8	2.78x10^10	7.98x10^6	429.77	2.73x10^-11	9.29x10^-11	2.87
198	5.37x10^8	5.04x10^10	1.73x10^7	429.77	2.73x10^-11	9.29x10^-11	2.87
199	3.12x10^8	2.50x10^10	7.31x10^6	429.77	2.73x10^-11	9.29x10^-11	2.87
200	2.34x10^8	1.72x10^10	7.90x10^6	429.77	2.73x10^-11	9.29x10^-11	2.87
201	6.21x10^8	6.07x10^10	1.95x10^7	429.77	2.73x10^-11	9.29x10^-11	2.87
202	2.06x10^8	1.46x10^10	7.93x10^6	429.77	2.73x10^-11	9.29x10^-11	2.87
203	6.44x10^8	6.36x10^10	1.92x10^7	429.77	2.73x10^-11	9.29x10^-11	2.87
204	3.18x10^8	2.56x10^10	6.57x10^6	429.77	2.73x10^-11	9.29x10^-11	2.87
205	6.15x10^8	6.00x10^10	4.29x10^7	429.77	2.73x10^-11	9.29x10^-11	2.87
206	8.56x10^8	9.20x10^10	4.87x10^7	464.21	2.26x10^-11	7.67x10^-11	2.76
207	1.01x10^9	1.14x10^11	1.93x10^7	464.21	2.26x10^-11	7.67x10^-11	2.76
208	1.19x10^9	1.41x10^11	1.87x10^7	464.21	2.26x10^-11	7.67x10^-11	2.76
209	1.21x10^9	1.44x10^11	4.69x10^7	498.66	1.89x10^-11	6.42x10^-11	2.66

Results

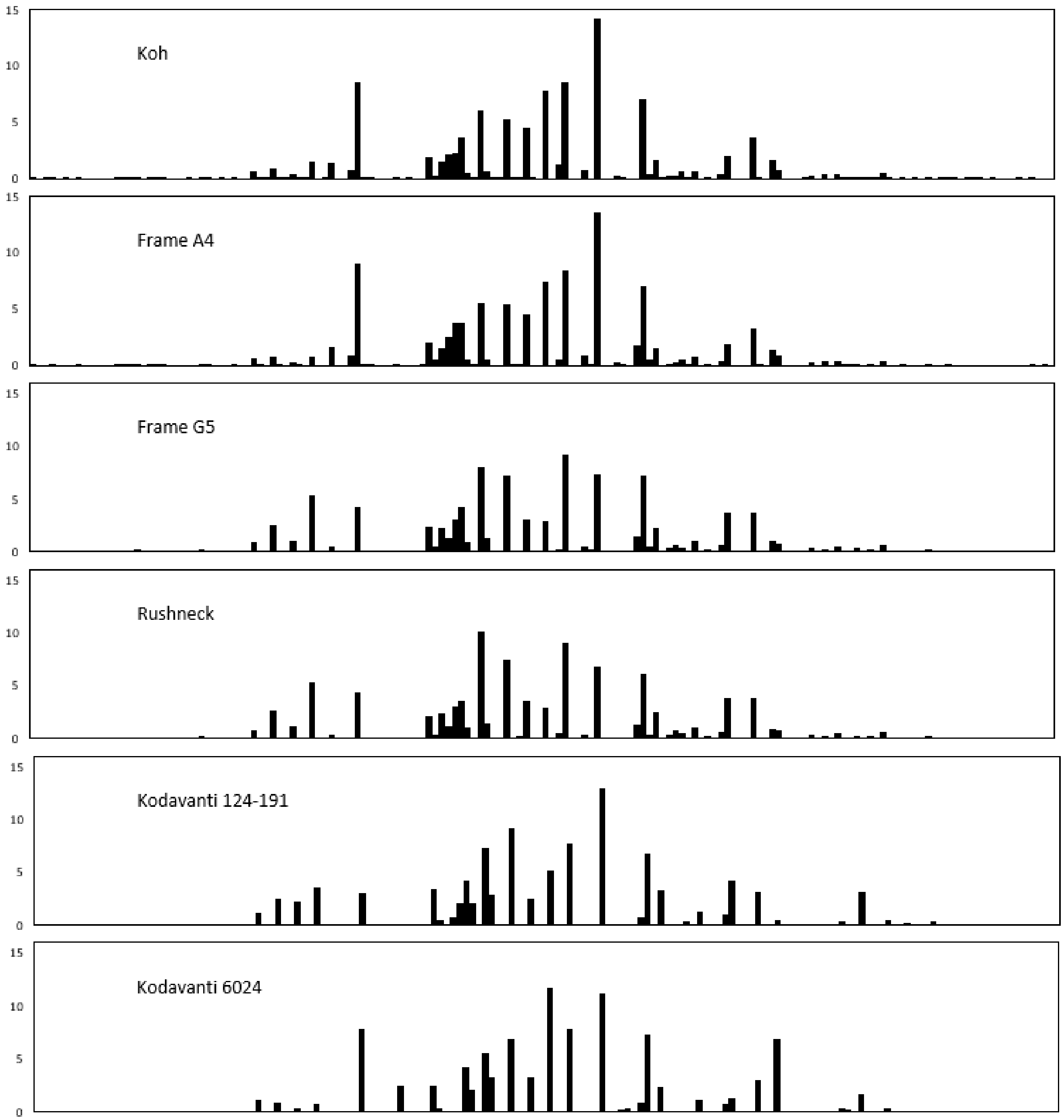

Figure S3. Comparison of different Aroclor 1254 profiles reported by different laboratories. Aroclor 1254 congener distributions are variable depending on the lot.

	Air	Air	Brick Wall	Air	Cinder-block Wall	Dry Wall	Tile	Air	Air	Cinder-blcok Wall	Air	Dry Wall	Carpet	Carpet	Glass Blocks	Cove Base	Carpet	Carpet	Carpet	Carpet	Carpet	Carpet	Carpet
Predicted Emissions 1254	0.970	0.969	0.967	0.967	0.966	0.964	0.958	0.956	0.950	0.948	0.947	0.936	0.916	0.913	0.908	0.894	0.885	0.885	0.882	0.871	0.816	0.814	0.814
Rushneck 1254	0.810	0.798	0.774	0.793	0.781	0.761	0.757	0.768	0.755	0.740	0.754	0.716	0.695	0.681	0.831	0.633	0.643	0.641	0.630	0.620	0.584	0.587	0.576
1254 G4	0.791	0.780	0.755	0.775	0.760	0.743	0.735	0.749	0.736	0.720	0.734	0.697	0.680	0.667	0.801	0.620	0.630	0.627	0.619	0.609	0.576	0.581	0.567
K1248	0.726	0.734	0.749	0.731	0.746	0.776	0.745	0.736	0.730	0.757	0.730	0.746	0.761	0.755	0.665	0.723	0.784	0.740	0.751	0.726	0.684	0.683	0.676
1248 A3.5	0.711	0.719	0.734	0.716	0.732	0.762	0.730	0.722	0.715	0.743	0.715	0.731	0.747	0.741	0.655	0.708	0.770	0.726	0.737	0.711	0.671	0.670	0.663
Rushneck 1248	0.687	0.697	0.714	0.693	0.710	0.742	0.710	0.697	0.691	0.721	0.690	0.708	0.724	0.718	0.636	0.683	0.747	0.703	0.714	0.688	0.646	0.645	0.639
1248 G3.5	0.671	0.678	0.693	0.674	0.693	0.723	0.691	0.681	0.673	0.702	0.674	0.688	0.704	0.696	0.642	0.653	0.727	0.675	0.685	0.658	0.612	0.611	0.603
Lot124-191	0.699	0.687	0.658	0.681	0.666	0.646	0.641	0.658	0.644	0.625	0.645	0.603	0.588	0.572	0.722	0.525	0.537	0.532	0.523	0.506	0.489	0.493	0.477
K1254	0.561	0.550	0.529	0.543	0.530	0.522	0.507	0.516	0.498	0.489	0.498	0.461	0.455	0.441	0.615	0.382	0.410	0.399	0.394	0.384	0.365	0.370	0.354
1254 A4	0.544	0.532	0.511	0.525	0.513	0.505	0.489	0.498	0.480	0.471	0.479	0.442	0.437	0.422	0.611	0.360	0.391	0.379	0.373	0.363	0.344	0.349	0.333
Lot 6024	0.526	0.514	0.491	0.506	0.496	0.485	0.475	0.485	0.468	0.455	0.469	0.430	0.423	0.410	0.586	0.352	0.380	0.368	0.361	0.351	0.334	0.340	0.323
Rushneck 1242	0.429	0.439	0.452	0.438	0.449	0.487	0.441	0.454	0.453	0.486	0.456	0.479	0.531	0.515	0.361	0.495	0.601	0.529	0.554	0.532	0.556	0.562	0.539
1242 A3	0.423	0.432	0.443	0.432	0.441	0.478	0.432	0.449	0.448	0.478	0.451	0.473	0.526	0.510	0.353	0.492	0.596	0.525	0.550	0.529	0.558	0.565	0.541
K1242	0.413	0.423	0.435	0.423	0.432	0.470	0.424	0.441	0.440	0.471	0.443	0.467	0.520	0.504	0.340	0.487	0.593	0.520	0.546	0.526	0.555	0.562	0.538
1242 S3B	0.414	0.423	0.434	0.423	0.432	0.469	0.423	0.440	0.440	0.470	0.443	0.464	0.518	0.501	0.347	0.485	0.588	0.516	0.542	0.522	0.558	0.566	0.541
1242 G3	0.390	0.399	0.411	0.399	0.409	0.447	0.400	0.418	0.417	0.449	0.421	0.444	0.498	0.482	0.320	0.463	0.572	0.498	0.524	0.503	0.525	0.531	0.507
K1016	0.298	0.310	0.323	0.313	0.318	0.357	0.310	0.332	0.335	0.367	0.337	0.368	0.427	0.414	0.186	0.411	0.514	0.443	0.473	0.454	0.493	0.500	0.476
1016 A2	0.259	0.271	0.284	0.275	0.278	0.315	0.269	0.293	0.299	0.328	0.300	0.331	0.391	0.378	0.137	0.381	0.480	0.412	0.443	0.423	0.468	0.475	0.451
1016 S2	0.257	0.270	0.282	0.273	0.276	0.314	0.267	0.292	0.297	0.326	0.298	0.330	0.390	0.376	0.135	0.380	0.478	0.411	0.442	0.422	0.467	0.474	0.450
Rushneck 1016	0.256	0.268	0.280	0.272	0.276	0.313	0.266	0.290	0.295	0.325	0.296	0.328	0.387	0.373	0.137	0.376	0.475	0.408	0.439	0.417	0.462	0.469	0.445
Rushneck 1260	0.275	0.273	0.237	0.271	0.243	0.223	0.225	0.246	0.246	0.225	0.242	0.218	0.210	0.192	0.266	0.173	0.177	0.177	0.172	0.172	0.169	0.176	0.162
Rushneck 1232	0.223	0.229	0.231	0.231	0.231	0.254	0.221	0.241	0.245	0.259	0.246	0.255	0.304	0.290	0.179	0.300	0.354	0.313	0.340	0.326	0.522	0.549	0.525
1260 A5	0.246	0.244	0.209	0.242	0.214	0.196	0.196	0.217	0.218	0.197	0.214	0.191	0.185	0.168	0.234	0.150	0.155	0.154	0.151	0.150	0.149	0.157	0.143
1260 S5	0.243	0.241	0.206	0.239	0.211	0.193	0.193	0.214	0.215	0.194	0.211	0.189	0.182	0.165	0.230	0.148	0.152	0.152	0.148	0.148	0.147	0.155	0.140
1232 A1.5	0.193	0.198	0.199	0.200	0.199	0.220	0.189	0.210	0.215	0.225	0.215	0.223	0.270	0.256	0.149	0.272	0.317	0.280	0.307	0.294	0.504	0.532	0.509
1232 G1.5	0.190	0.195	0.196	0.197	0.196	0.217	0.186	0.207	0.212	0.222	0.212	0.220	0.266	0.253	0.147	0.269	0.313	0.277	0.303	0.291	0.503	0.533	0.509
1260 G5	0.223	0.222	0.188	0.221	0.193	0.175	0.175	0.196	0.196	0.176	0.192	0.171	0.166	0.149	0.209	0.133	0.136	0.137	0.134	0.133	0.133	0.140	0.127
Rushneck 1262	0.139	0.140	0.118	0.139	0.120	0.110	0.108	0.123	0.124	0.112	0.121	0.109	0.108	0.096	0.123	0.087	0.090	0.090	0.089	0.089	0.090	0.094	0.085
1262 G6	0.124	0.124	0.102	0.123	0.104	0.094	0.093	0.107	0.108	0.095	0.105	0.093	0.091	0.080	0.108	0.072	0.074	0.074	0.074	0.074	0.076	0.080	0.071
1262 A6	0.120	0.121	0.100	0.120	0.102	0.093	0.091	0.105	0.106	0.095	0.103	0.093	0.091	0.081	0.104	0.073	0.076	0.075	0.075	0.075	0.077	0.081	0.072
K1221	0.039	0.041	0.037	0.043	0.039	0.044	0.032	0.046	0.051	0.048	0.050	0.047	0.072	0.066	0.025	0.091	0.089	0.084	0.102	0.096	0.342	0.374	0.362
1221 A1	0.029	0.031	0.026	0.033	0.028	0.033	0.021	0.035	0.041	0.037	0.039	0.037	0.062	0.056	0.015	0.082	0.079	0.075	0.092	0.087	0.331	0.363	0.350
Rushneck 1221	0.010	0.012	0.008	0.013	0.011	0.011	0.006	0.013	0.017	0.012	0.015	0.011	0.021	0.021	0.007	0.039	0.027	0.031	0.043	0.038	0.265	0.295	0.292
Rushneck 1268	0.002	0.002	0.002	0.002	0.002	0.001	0.001	0.001	0.001	0.001	0.001	0.001	0.001	0.001	0.001	0.001	0.001	0.001	0.001	0.001	0.003	0.001	0.002
Room	203	203	203	203	203	203	203	205	205	205	205	205	205	205	203	203	205	201	201	201	203	203	203

Figure S4. Cosine theta similarity results for all samples and Aroclors.

	Air	Air	Brick Wall	Air	Cinder-block Wall	Dry Wall	Tile	Air	Air	Cinder-block Wall	Air	Dry Wall	Carpet	Carpet	Glass Blocks	Cove Base	Carpet	Carpet	Carpet	Carpet	Carpet	Carpet	Carpet
v-Kodavanti Lot 124-191	0.929	0.935	0.941	0.936	0.937	0.941	0.940	0.934	0.936	0.934	0.932	0.933	0.910	0.916	0.804	0.927	0.892	0.899	0.901	0.863	0.830	0.826	0.833
v-Rushneck 1254	0.920	0.933	0.945	0.937	0.930	0.944	0.935	0.931	0.940	0.943	0.933	0.948	0.936	0.945	0.736	0.979	0.936	0.948	0.960	0.947	0.924	0.924	0.929
v-Frame 1254 G4	0.898	0.911	0.923	0.915	0.908	0.925	0.911	0.911	0.921	0.924	0.913	0.931	0.925	0.933	0.705	0.970	0.932	0.940	0.955	0.944	0.923	0.924	0.926
v-Koh 1254	0.835	0.836	0.843	0.832	0.839	0.852	0.837	0.824	0.815	0.825	0.813	0.807	0.799	0.795	0.829	0.763	0.778	0.763	0.765	0.750	0.728	0.731	0.728
v-Kodavanti Lot 6024	0.785	0.777	0.776	0.768	0.785	0.786	0.782	0.767	0.751	0.756	0.755	0.733	0.717	0.708	0.857	0.651	0.679	0.658	0.648	0.632	0.579	0.577	0.574
v-Frame 1254 A4	0.754	0.750	0.752	0.743	0.755	0.765	0.749	0.737	0.723	0.734	0.725	0.710	0.707	0.695	0.812	0.646	0.680	0.656	0.654	0.641	0.618	0.621	0.614
v-Frame 1260 G5	0.703	0.694	0.659	0.691	0.677	0.644	0.660	0.679	0.676	0.649	0.678	0.640	0.611	0.589	0.740	0.561	0.556	0.553	0.537	0.536	0.521	0.526	0.516
v-Rushneck 1260	0.676	0.670	0.640	0.669	0.655	0.636	0.638	0.667	0.668	0.648	0.672	0.643	0.642	0.616	0.673	0.617	0.622	0.608	0.607	0.604	0.677	0.691	0.668
v-Frame 1260 A5	0.642	0.635	0.603	0.634	0.619	0.599	0.600	0.630	0.631	0.609	0.635	0.603	0.602	0.576	0.650	0.574	0.579	0.566	0.565	0.562	0.656	0.674	0.651
v-Frame 1260 S5	0.635	0.628	0.595	0.627	0.612	0.591	0.592	0.623	0.623	0.601	0.627	0.595	0.595	0.569	0.646	0.566	0.572	0.558	0.558	0.554	0.650	0.668	0.644
v-Koh 1248	0.369	0.381	0.392	0.385	0.384	0.419	0.378	0.399	0.406	0.427	0.406	0.433	0.483	0.472	0.239	0.491	0.556	0.508	0.538	0.519	0.574	0.581	0.560
v-Frame 1248 G3.5	0.364	0.375	0.389	0.378	0.383	0.419	0.377	0.394	0.397	0.425	0.399	0.427	0.474	0.463	0.255	0.459	0.549	0.487	0.514	0.490	0.497	0.498	0.481
v-Rushneck 1248	0.354	0.367	0.377	0.370	0.370	0.405	0.364	0.384	0.390	0.412	0.390	0.417	0.466	0.455	0.233	0.470	0.538	0.489	0.519	0.497	0.548	0.553	0.533
v-Frame 1248 A3.5	0.345	0.357	0.366	0.361	0.360	0.393	0.353	0.375	0.381	0.401	0.382	0.407	0.457	0.445	0.225	0.463	0.528	0.480	0.510	0.490	0.548	0.554	0.533
v-Frame 1262 G6	0.351	0.352	0.326	0.352	0.335	0.332	0.314	0.350	0.356	0.344	0.358	0.343	0.372	0.346	0.328	0.364	0.385	0.363	0.378	0.372	0.541	0.566	0.534
v-Rushneck 1262	0.270	0.274	0.257	0.275	0.261	0.270	0.244	0.279	0.286	0.283	0.288	0.285	0.327	0.303	0.220	0.330	0.362	0.333	0.354	0.349	0.488	0.507	0.473
v-Frame 1262 A6	0.256	0.259	0.241	0.261	0.246	0.253	0.228	0.264	0.271	0.266	0.272	0.268	0.309	0.284	0.213	0.311	0.341	0.313	0.334	0.328	0.481	0.503	0.469
v-Rushneck 1242	0.094	0.098	0.092	0.101	0.093	0.111	0.081	0.111	0.118	0.123	0.121	0.127	0.179	0.161	0.040	0.196	0.233	0.202	0.228	0.222	0.386	0.406	0.373
v-Frame 1242 G3	0.093	0.097	0.092	0.100	0.092	0.111	0.080	0.111	0.118	0.124	0.121	0.128	0.183	0.163	0.038	0.196	0.239	0.204	0.231	0.225	0.375	0.394	0.361
v-Rushneck 1268	0.102	0.105	0.105	0.105	0.104	0.111	0.101	0.108	0.109	0.113	0.109	0.114	0.127	0.122	0.074	0.127	0.141	0.130	0.138	0.133	0.158	0.160	0.154
v-Frame 1242 A3	0.090	0.094	0.088	0.097	0.088	0.106	0.077	0.107	0.114	0.118	0.116	0.122	0.175	0.156	0.038	0.191	0.227	0.197	0.223	0.217	0.379	0.399	0.366
v-Koh 1242	0.089	0.094	0.088	0.096	0.088	0.106	0.076	0.106	0.113	0.118	0.116	0.121	0.174	0.155	0.037	0.191	0.226	0.196	0.222	0.216	0.382	0.402	0.370
v-Koh 1016	0.083	0.088	0.082	0.090	0.082	0.100	0.070	0.101	0.108	0.113	0.110	0.117	0.170	0.151	0.027	0.187	0.223	0.192	0.219	0.213	0.375	0.395	0.363
v-Frame 1242 S3B	0.084	0.088	0.081	0.091	0.082	0.099	0.070	0.100	0.107	0.111	0.109	0.114	0.166	0.147	0.035	0.184	0.215	0.187	0.213	0.208	0.380	0.401	0.369
v-Rushneck 1016	0.081	0.086	0.080	0.089	0.080	0.098	0.068	0.099	0.106	0.111	0.109	0.115	0.169	0.150	0.024	0.186	0.225	0.193	0.220	0.214	0.374	0.393	0.360
v-Frame 1016 A2	0.081	0.085	0.079	0.088	0.080	0.098	0.067	0.099	0.106	0.111	0.108	0.115	0.169	0.150	0.024	0.186	0.224	0.192	0.219	0.213	0.371	0.390	0.358
v-Frame 1016 S2	0.080	0.085	0.079	0.088	0.079	0.097	0.067	0.098	0.105	0.110	0.108	0.114	0.168	0.149	0.024	0.185	0.223	0.191	0.218	0.212	0.372	0.391	0.358
v-Rushneck 1232	0.032	0.035	0.028	0.037	0.030	0.037	0.022	0.040	0.047	0.043	0.046	0.043	0.075	0.066	0.012	0.099	0.098	0.091	0.112	0.106	0.366	0.399	0.379
v-Frame 1232 A1.5	0.030	0.032	0.025	0.034	0.027	0.034	0.019	0.037	0.043	0.039	0.043	0.040	0.069	0.061	0.011	0.094	0.091	0.086	0.105	0.100	0.360	0.394	0.374
v-Frame 1232 G1.5	0.029	0.032	0.025	0.034	0.027	0.033	0.019	0.036	0.043	0.038	0.042	0.039	0.068	0.060	0.010	0.094	0.089	0.085	0.104	0.099	0.361	0.394	0.376
v-Frame 1221 A1	0.015	0.016	0.011	0.019	0.013	0.016	0.007	0.019	0.025	0.019	0.023	0.019	0.039	0.034	0.005	0.062	0.051	0.052	0.068	0.064	0.313	0.346	0.334
v-Koh 1221	0.014	0.016	0.011	0.018	0.013	0.016	0.007	0.019	0.024	0.019	0.023	0.018	0.037	0.033	0.005	0.060	0.049	0.050	0.066	0.061	0.312	0.345	0.334
v-Rushneck 1221	0.007	0.009	0.005	0.010	0.007	0.008	0.003	0.010	0.014	0.009	0.012	0.008	0.018	0.018	0.004	0.036	0.024	0.028	0.040	0.035	0.263	0.293	0.290
Room	203	203	203	203	203	203	203	205	205	205	205	205	205	205	203	203	205	201	201	201	203	203	203

Figure S5. Cosine theta similarity results for all samples and vaporized Aroclors. Vaporized Aroclor profiles were determined by multiplying the Aroclor profiles by each congener's vapor pressure and normalized to the sum of all congeners.[5,6]

References

(1) Hua, J. B. X., Marek, R.F., Hornbuckle, K.C. Dataset for Polyurethane Foam Emission Samplers to Identify Sources of Airborne Polychlorinated Biphenyls (PCBs) from Glass Block Windows and Other Room Surfaces in a Vermont School 2023. DOI: 10.25820/data.006632
(2) Shoeib, M.; Harner, T. Characterization and Comparison of Three Passive Air Samplers for Persistent Organic Pollutants. *Environmental Science & Technology* **2002**, *36* (19), 4142-4151. DOI: 10.1021/es020635t.
(3) Herkert, N. J.; Martinez, A.; Hornbuckle, K. C. A Model Using Local Weather Data to Determine the Effective Sampling Volume for PCB Congeners Collected on Passive Air Samplers. *Environmental Science & Technology* **2016**, *50* (13), 6690-6697. DOI: 10.1021/acs.est.6b00319.
(4) Jahnke, J. C.; Hornbuckle, K. C. PCB Emissions from Paint Colorants. *Environmental Science & Technology* **2019**, *53* (9), 5187-5194. DOI: 10.1021/acs.est.9b01087.
(5) Rodenburg, L. A.; Du, S.; Xiao, B.; Fennell, D. E. Source apportionment of polychlorinated biphenyls in the New York/New Jersey Harbor. *Chemosphere* **2011**, *83* (6), 792-798. DOI: 10.1016/j.chemosphere.2011.02.058.
(6) Fischer, R. C.; Wittlinger, R.; Ballschmiter, K. Retention-index based vapor pressure estimation for polychlorobiphenyl (PCB) by gas chromatography. *Fresenius' Journal of Analytical Chemistry* **1992**, *342* (4), 421-425. DOI: 10.1007/BF00322199.
